# Glucocorticoids in relation to behavior, morphology, and physiology as proxy indicators for the assessment of animal welfare. A systematic mapping review

**DOI:** 10.3389/fvets.2022.954607

**Published:** 2023-01-06

**Authors:** Inga Tiemann, Lisa B. Fijn, Marc Bagaria, Esther M. A. Langen, F. Josef van der Staay, Saskia S. Arndt, Cathalijn Leenaars, Vivian C. Goerlich

**Affiliations:** ^1^Faculty of Agriculture, Institute of Agricultural Engineering, University of Bonn, Bonn, Germany; ^2^Division of Animals in Science and Society, Department of Population Health Sciences, Faculty of Veterinary Medicine, Utrecht University, Utrecht, Netherlands; ^3^Division of Farm Animal Health, Behaviour and Welfare Group, Department of Population Health Sciences, Faculty of Veterinary Medicine, Utrecht University, Utrecht, Netherlands; ^4^Institute for Laboratory Animal Science, Hannover Medical School, Hanover, Germany

**Keywords:** welfare proxy, readout parameters, endocrine biomarkers, hormone metabolites, stress, animal husbandry, systematic review, welfare indicator

## Abstract

Translating theoretical concepts of animal welfare into quantitative assessment protocols is an ongoing challenge. Glucocorticoids (GCs) are frequently used as physiological measure in welfare assessment. The interpretation of levels of GCs and especially their relation to welfare, however, is not as straightforward, questioning the informative power of GCs. The aim of this systematic mapping review was therefore to provide an overview of the relevant literature to identify global patterns in studies using GCs as proxy for the assessment of welfare of vertebrate species. Following a systematic protocol and a-priory inclusion criteria, 509 studies with 517 experiments were selected for data extraction. The outcome of the experiments was categorized based on whether the intervention significantly affected levels of GCs, and whether these effects were accompanied by changes in behavior, morphology and physiology. Additional information, such as animal species, type of intervention, experimental set up and sample type used for GC determination was extracted, as well. Given the broad scope and large variation in included experiments, meta-analyses were not performed, but outcomes are presented to encourage further, in-depth analyses of the data set. The interventions did not consistently lead to changes in GCs with respect to the original authors hypothesis. Changes in GCs were not consistently paralleled by changes in additional assessment parameter on behavior, morphology and physiology. The minority of experiment quantified GCs in less invasive sample matrices compared to blood. Interventions showed a large variability, and species such as fish were underrepresented, especially in the assessment of behavior. The inconclusive effects on GCs and additional assessment parameter urges for further validation of techniques and welfare proxies. Several conceptual and technical challenges need to be met to create standardized and robust welfare assessment protocols and to determine the role of GCs herein.

## 1. Introduction

Safeguarding and improving the welfare of animals under human care, irrespective of species and context, is a goal recognized by science and society. Many theoretical frameworks have been put forward to conceptualize what “good” welfare is and how animal welfare could be quantified. These concepts lay the basis for practical recommendation for assessing animal welfare and measures for improving animal welfare [e.g., (1)]. The concept of the “Five Freedoms,” proposed by the Brambell committee in 1965 (2), is the earliest and most influential approach to defining the basic aspects of husbandry necessary to safeguard welfare especially of farmed animals. Meanwhile, next to the mere absence of negative states, the importance of the inclusion of positive states in welfare has been emphasized (3). Current concepts incorporate several domains, such as behavior, naturalness, health, and physiology [e.g., the “Five Domains” concept; (4, 5)]. In this review, we use the following conceptual approach to animal welfare: animal welfare is a dynamic process, not a momentary snapshot, to which both positive and negative states contribute (3, 6). The ability to cope and adapt to environmental stimuli and stressors, delimited by the animal's adaptive capacity, is the basis for the animal to “[…] reach a state that it perceives as positive […]” (6). The mental and emotional state of an animal, which is accompanied by correlated physiological patterns, therefore forms a crucial part of welfare (7–9). Establishing the potential relation between these aspects, however, needs further research.

While many different concepts contribute valuable insight into animal welfare, the assessment of welfare is an ongoing challenge. The identification of measurements concerning health, behavior, and/or physiology to derive readout parameters indicative of a positive, or negative, welfare state is a much-debated goal in animal welfare research (10). Ultimately, if one or a few well-validated parameters would correlate highly with other parameters that are considered valid proxies/biomarkers of animal welfare, these could serve as index of welfare [iceberg indicators, e.g., (11)]. In this case, one could dispense with a multitude of measurements in animal welfare research and focus on few key indicators.

Despite critical evaluations of the usefulness of GC values as a proxy indicator for stress (12) and welfare states (13–17), the assumption of animals exhibiting high levels of GCs, and therefore experiencing a diminished welfare, remains widespread (18). Given that welfare is a multidimensional concept, it should therefore be assessed using a combination of behavioral, morphological, and physiological indicators (14). A broad range of additional parameters has been investigated as potential proxy indicators of animal welfare. Some may be measurable directly and quantitatively, e.g., the presence of wounds or infections, others can only be inferred, such as subjective mental states and cognitive bias (19).

In search for key indicators of animal welfare, the measurement of a physiological parameter may imply objectiveness and straightforward interpretation. Glucocorticoids (GCs), in particular the steroid hormones cortisol and corticosterone, have gained much popularity in research on welfare of vertebrate species. External and internal stimuli and stressors may affect an individual's welfare, and an individual's welfare state may affect its ability to cope with these. GCs mediate the endocrine stress response, which is orchestrated by the hypothalamus–pituitary–adrenal (HPA) axis in mammals, birds and reptiles, and the hypothalamic–pituitary–interrenal (HPI) axis in fish and amphibians (20). Notably, next to being a key player in the endocrine response to stressors, GCs induce a manifold of behavioral and physiological processes to promote restoring homeostasis and survival (21). Given the pleiotropic actions of GCs, the interpretation of GC levels and release patterns proves complex (12, 21–23). GCs may rise not only in response to a stressor with potentially negative consequences, but also in response to stimuli such as environmental enrichment or sexual encounters (13, 24–26). Notably, the interpretation of the valence of the stimulus, whether it is perceived as positive or as negative and potentially threatful, may depend on the individual's personality and cognitive traits (18, 27, 28). Adding onto the biological complexity of interpreting GC levels, are the variations in techniques to sample and determine GC levels in various tissues. To make robust assumptions on the relation between welfare and GCs, methodological limitations need to be identified and overcome.

Welfare is dynamic and describes the individual's coping with stimuli and stressors (14). In combination, GC levels may add information on the activation of the HPA axis and arousal, aiding the interpretation of an animal's response to an intervention aimed at affecting welfare. The usefulness of GCs in assessing an animal's welfare may very well-depend on the time frame within which GC levels are monitored. GCs are time-sensitive in their excretion after a triggering event, but also with regard to biological rhythms (29). GCs excretion follows ultradian, circadian and seasonal rhythms, leading to measurable variation in levels under undisturbed circumstances (14, 30, 31). Given the circadian rhythm, an animal should ideally be monitored for at least 24 h to infer information on deviation in GC release (32). Moreover, the genomic actions of GCs need several hours to come into action (21). Finally, considering the central role of the animal's ability to cope and adapt in welfare concepts, monitoring should ideally last longer than 24 h.

Given the popularity, and criticism, of GC measurements in welfare assessment, we performed a mapping review of the research field to identify general trends and provide a basis for future, in-depth analyses. A mapping review “is a high-level review with a broad research question and presents the global results” (33). It follows a systematic search of literature and data extraction, but does not provide detailed information on a meta-analysis level. Rather, the results aid the identification of trends and gaps in knowledge and suggests avenues for future studies.

Based on strict a-priori criteria, we examined experimental studies which tested an intervention aimed at affecting the welfare of a target population. We were interested in the consistency of patterns of GCs, and welfare read out parameters in the domains behavior, morphology, and physiology, in welfare assessment studies. Following our conceptual approach to welfare being a multidimensional construct, we selected studies which measured GCs and concurrently parameters related to behavior, morphology, and physiology to evaluate the effects of interventions (15). Moreover, as we see welfare as being dynamic and comprising more than the peak GC response to a stressor [which typically lasts < 24 h (30, 34), but may exert longer impact on the animal (32)], we restricted our search to studies following animals longer than 24 h after an acute intervention or studies that investigated long-term interventions.

From the included studies, we extracted data on the effects of the intervention on GCs and behavior, morphology, and physiology. We then categorized the impact of the welfare intervention on the outcomes within the four domains into “no effect,” “homogenous effect,” and “heterogenous effect” (Table 1) to provide an overview of the outcome of interest in relation the study characteristics. These within-domain categorizations are presented next to each other to reflect patterns across readout parameters.

**Table 1:** Conceptual approach to animal welfare underlying the selection of studies included in this mapping review and categorization of parameter outcomes within the four domains GCs, behavior, morphology or physiology (B, M, P).

**Concept**	**Working definition**
Animal welfare	A dynamic process delimited by the animal's capacity to cope with the environment to reach a state that it perceives as positive (6).
Intervention effect (on GC and/or other parameters measured)	Yes: The original authors observed a statistically significant effect of the intervention on the readout parameters within the four domains (GCs, B, M, P) compared to baseline or the control group according to the original analyses. No: The original authors did not observe a significant effect of the intervention on the readout parameters.
Homogeneous effects	Significant and consistent effect of the welfare intervention on the readout parameters within the four domains (GCs, B, M, P); consistent effect refers to a distinct change in readout parameters compared to baseline/control irrespective of direction (increase or decrease). This term covers the specificity of GCs to show an acute response, i.e., an increase in time proximity to the intervention/event and a decrease afterwards.
Heterogeneous effects	Inconclusive or inconsistent effect of the welfare intervention on the readout parameters within the four domains (GCs, B, M, P); inconclusive effect refers to significant but ambiguous pattern in changes of the readout parameters compared to baseline/control; inconsistent effect refers to the occurrence of significant as well as non-significant changes of the readout parameters compared to baseline/control.

We expected GC levels to change in response to welfare interventions in congruence with changes in readout parameters related to behavior, morphology, and physiology (e.g., in both domains “behavior and “GCs” a homogenous effect). Moreover, we expected GC outcomes to follow the author hypothesis. To provide an overview of the experiments, we report general aspects of the experimental studies, such as animal species, experimental design, and sample matrix for GC determination, as well.

With more than 500 records included, we present one of the largest mapping reviews focusing on GC measurements in the field of animal welfare assessment. Our results provide an overview of the field of research on animal welfare, describing the available evidence on the relation between GCs, additional readout parameters and welfare of vertebrate species.

## 2. Material and methods

### 2.1. Literature search strategy

The research question leading to this mapping review was: Do GCs change consistently in response to a welfare intervention? For the comprehensive search strategy, the question was rephrased according to the PICO-format (35) to: do GCs (Outcome) consistently change due to interventions potentially affecting welfare (Intervention) compared to within- or between-subject control conditions (Condition) in non-human vertebrate animals (Population)?

Therefore, this mapping review is based on a comprehensive search strategy, using multiple databases: PubMed, Embase and Web of Science. The searches for each database consisted of three search components [SCs, (36)]: SC1 welfare; SC2 glucocorticoids; SC3 all non-human vertebrates. The detailed search strings covered synonyms, alternative spellings and related terms, such as wellbeing for welfare.

For PubMed and Embase searches, both thesaurus-terms (MeSH for PubMed and Emtree for Embase) and title/abstract/keywords terms were included (Web of Science does not use an internal thesaurus). SC1 (animal welfare) covered the terms animal welfare and animal wellbeing, SC2 (GCs) was adapted from Leenaars et al. (33), SC3 (animals) from the Syrcle animal filter (37), with invertebrates removed, and some terms added (title-abstract terms for additional avian species, e.g., “turkey”). The complete search strings can be found in the Supplementary material 1. All searches were performed in June 2020.

### 2.2. Study selection

Search results were imported into the reference manager Zotero, where duplicate and triplicate records were removed. All remaining records were imported into Rayyan QCRI (https://www.rayyan.ai/) for screening (38, 39).

In the following sections we refer to “records” as the reference to a study or book, etc., “report” and “study” as the published research paper, and “experiment” to the independent interventions investigated within a study.

The reviewer team (five people) were first trained to apply the inclusion and exclusion criteria consistently, using a set of 10 randomly chosen studies from the retrieved records. The retrieved reports were then screened for relevance in two steps: first title and abstract, then full text. Reports were sorted on title and allocated randomly to the reviewers. Screening of each report took place by at least two independent reviewers. Discrepancies were solved by discussion among the team based on the conceptual approach detailed in Table 1.

### 2.3. Inclusion and exclusion criteria

We included original research publications reporting the assessment of GCs and welfare in non-human vertebrates. We used the following exclusion criteria in title-abstract screening: (1) invertebrate or human study population, (2) no glucocorticoids measured in the study, (3) wrong publication type (no primary data, reviews, theses, conference abstracts), (4) animal welfare not primary focus. During full text screening we used the same exclusion criteria, in addition: (5) lack of an appropriate control for the intervention, and (6) non-English publication. We did not apply any publication date restriction.

The exclusion criterion (4) “animal welfare not primary focus” was operationalized as follows: studies needed to examine parameters of at least one additional domain (behavior, morphology, physiology) next to GCs, as welfare is more than purely the peak GC response to a stressor. As welfare is dynamic and should be monitored over a longer timeframe, we included only studies that followed the animals at least 24 h post intervention. These studies either investigated the long-term response to a short-term intervention, or the effects of a long-term intervention (chronic and/or repeated). Of these studies, all data covering GC, behavioral, morphological, and physiological measurements, thus also those collected within the first 24 h, were taken into consideration. Studies investigating welfare-related parameters over a period of < 24 h after the intervention were excluded.

The “appropriate control” could either be a pre-intervention baseline in within-subject experimental designs, or a separate group of animals not exposed to the intervention. Studies comparing groups without a clear control group (e.g., studies comparing different housing densities or diets where none was explicitly designated as control by the authors) were excluded.

### 2.4. Data extraction and analyses

From the final set of 509 included studies, we extracted the domains intervention, animals, sampling, and outcome. In total, 17 different categories of information were extracted per experiment included, such as numeric data (number of animals) and descriptive data [species, category, strain/breed (free text), and sex] for the domain “animals.” Other information categories covered descriptive data on the authors hypothesis, the type of welfare intervention, experimental design, sampling regime, and sample type used for determination of GCs (see Supplementary material 2). A priori, we agreed on a list of potential interventions, based on our experience with the field of research. If, during extraction, an intervention did not fit into one of these categories, it was labeled as “other.” If an intervention comprised several categories, it was labeled as “combination” and further specified in a free text column. The authors original hypothesis on the effect of the experimental intervention was classified as welfare *enhancing, diminishing*, or *no hypothesis*, based on agreement of at least three of the five reviewers.

Similarly, the impact of the intervention was scored for each readout parameter within the domains GCs, behavior, morphology, physiology (GCs, B, M, P). Based on agreement of at least three of the five reviewers, the outcome was scored as no effect or significant effect compared to baseline/control. Effects were scored as significant based on analytical statistics reported per experiment. The outcome within each domain was scored as homogenous if there was a consistent change in readout parameters compared to baseline/control, i.e., a distinct effect of the intervention on all readout parameters included in the statistical analyses. The outcome within each domain was scored as heterogenous if readout parameters showed inconclusive or inconsistent effects compared to baseline/control (see Table 1). We did not differentiate between an increase or decrease of single parameters, as we were interested in detecting changes in readout parameters caused by the intervention rather than judging on whether an increase or decrease of a readout parameter indicates an increase or decrease in welfare.

To avoid vote counting (40), statistical comparison of outcome categories within parameter domains (GCs, B, M, P) were not performed, but descriptive statistics are reported. The quantitative, but from a statistical point of view not analytical, description of the extracted information of the set of included studies was first organized in Excel (Microsoft Corporation, Redmond, WA, USA). Next, the percentage distribution was calculated for each result category and presented graphically (using SigmaPlot 14, Systat Software, San Jose, CA) or in tabular form. In general, n represents the number of studies and k the number of experiments included in the analyses.

We used VOSviewer for the exploratory visualization of the terms in the title and the abstracts (version 1.6.16; https://www.vosviewer.com/). The result are maps based on the co-occurrence of terms used in the titles and abstracts of the studies included. To simplify the set of terms, VOSviewer calculates clusters, indicated by distinct groups in the same color (41). The size of the circles and of the labels reflects the frequency of occurrence. Labels with low frequencies might not be shown to avoid overlapping. The relatedness is reflected as distance between two items and based on the VOS mapping technique using a similarity matrix. The total link strength indicates the sum of link strengths as a weight attribute, where the link strength is defined as number of links of an item with another item.

## 3. Results

### 3.1. Reference flow

**Figure 1:**
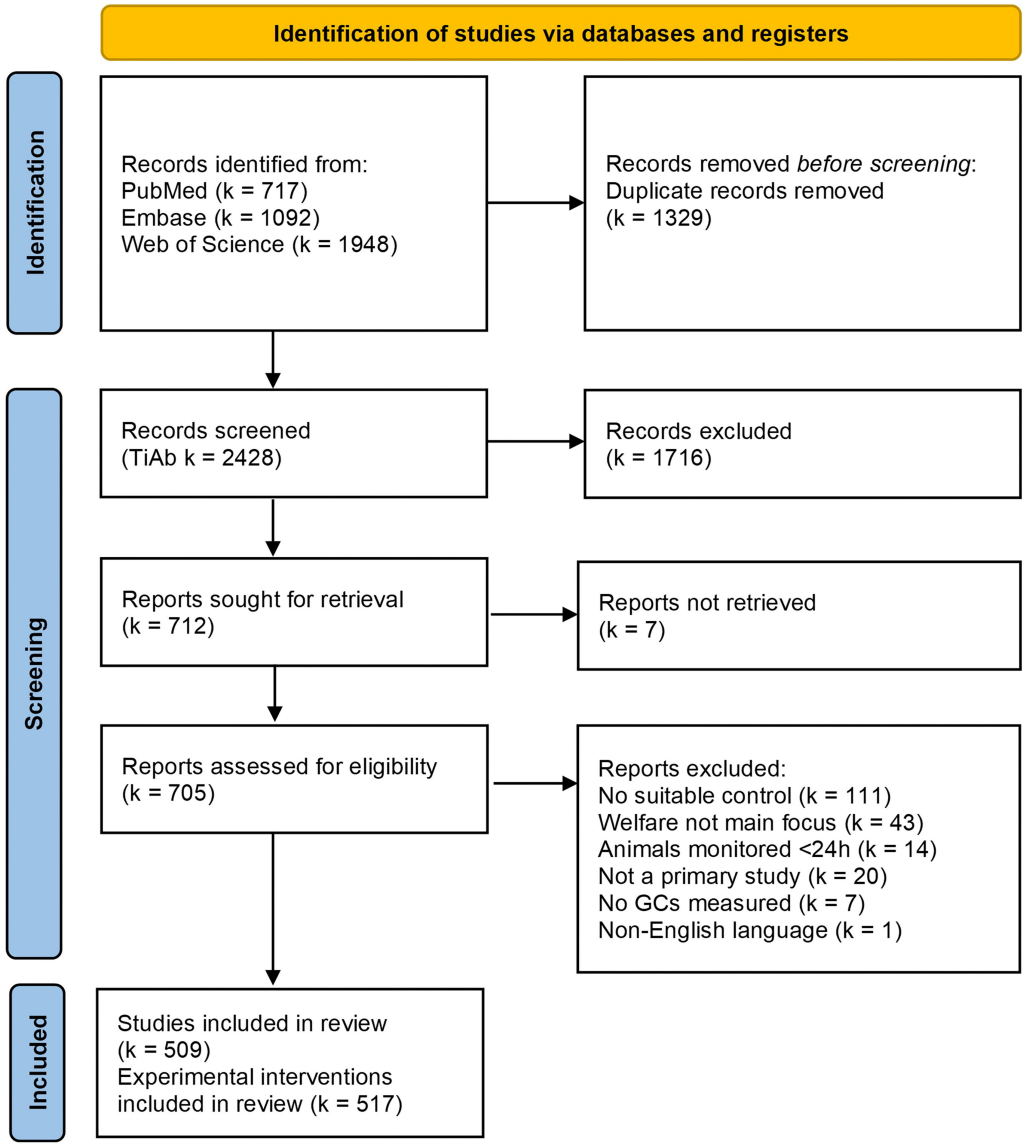
Modified PRISMA 2020 flow diagram of the mapping review detailing the database searches, the number of records screened for title and abstracts (TiAb) and full-text, and the final number of studies (*n*) and experiments [k] included for data extraction. Figure adapted from Page et al. (42).

Our literature searches provided 717 results from PubMed, 1,092 from Embase, and 1,948 from Web of Science. After duplicate removal, 2,428 records remained for title-abstract screening. After applying the exclusion criteria during title-abstract and subsequent full text screening, *n* = 509 studies were included in this mapping review [Figure 1, (42); see Supplementary material 3]. Four of these 509 studies, reported two independent experimental interventions, and one reported five independent experimental interventions, resulting in a total of k = 517 experiments for data extraction.

### 3.2. Network visualization

**Figure 2:**
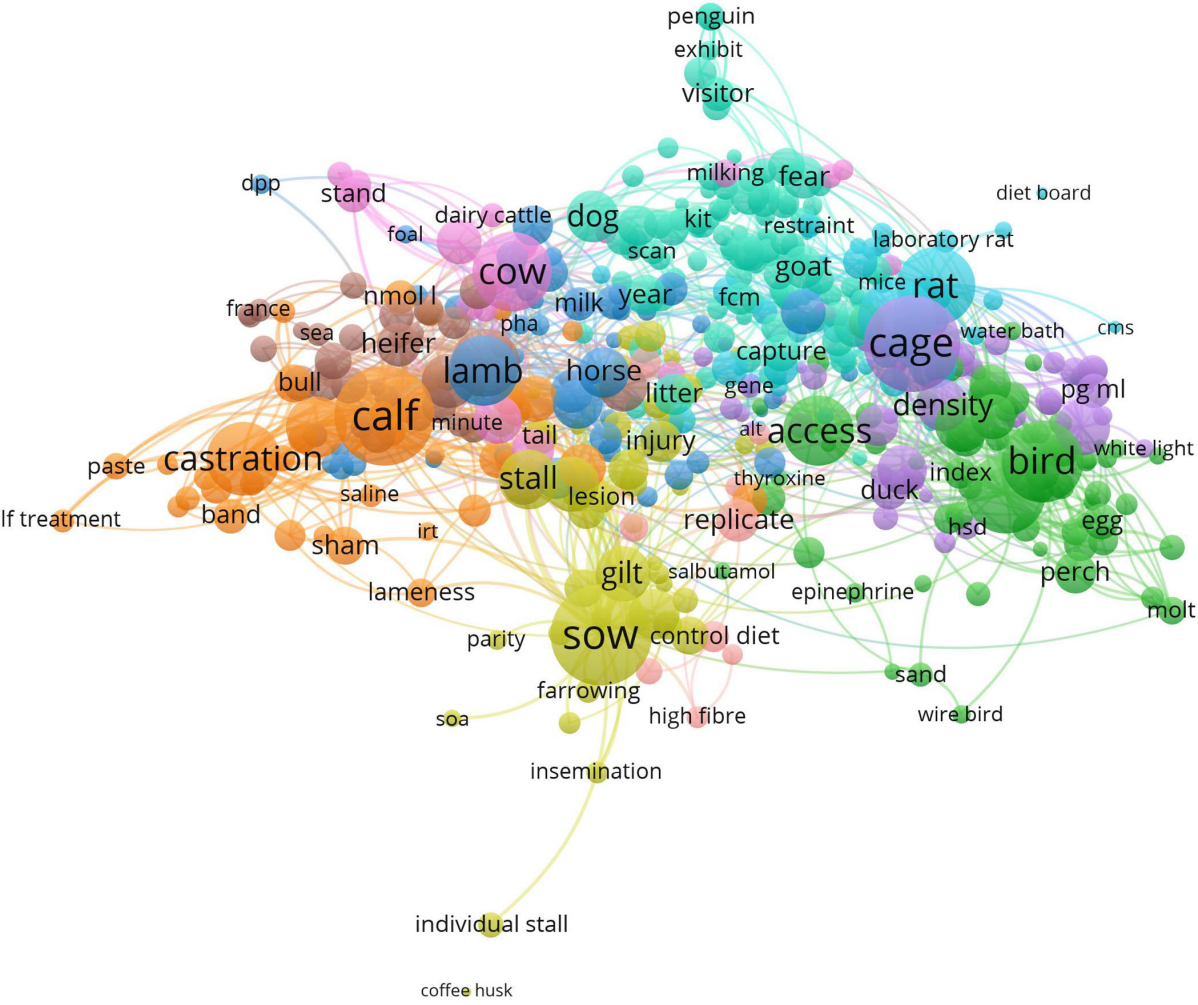
VOSviewer visualization of the abstract co-occurrence network based on the 509 studies included in this mapping review. Clusters are indicated by the same color. The frequency of occurrence of terms is reflected by the size of the circles. The distance between the circles reflects their relatedness based on the co-occurrence of the terms, which means the shorter the distance, the stronger the relatedness.

A link cloud was calculated based on the terms used in the abstracts of all included records (setting applied: min. occurrence of words: 7; resulting in items: 488; cluster: 9; links: 14,714; total link strength: 109,316; Figure 2). The main clusters revealed the different animal species investigated in the studies: pigs, cows, small rodents, and birds. The clusters also provide a first insight into the research topics predominantly investigated in these species. For pigs, the housing system as well as management including reproduction were frequently occurring themes. Comparable topics were relevant for cows, especially calves, although in this genus, the major focus was on castration. For mice and rats, research focused on the impact of housing and cage design on welfare. For birds, mainly chickens, the stocking density and husbandry system and their impact on welfare was commonly investigated. Research focused on zoo animals and their interaction with visitors is indicated by “penguin” and “exhibit.”

### 3.3. Effects on glucocorticoids

We scored whether the experimental intervention led to a statistically significant effect on GCs which could either be an increase or decrease of GC levels compared to control or pre-intervention baseline (homogenous effect) or a heterogenous effect (the intervention not leading to a clear and distinct change in GC levels, Table 1). Of the analyzed experiments, 39.26% (k = 203) found no significant effect of the intervention on GC levels, 38.10% (k = 197) found a homogenous effect and 22.63% (k = 117) of the experiments reported a heterogenous effect. Note that outcomes in which the GC response showed the typical course of an initial increase and following decrease were scored as homogenous.

### 3.4. Additional assessment parameters

In line with our conceptual approach to welfare, additional parameters from all three domains behavior, morphology, and physiology were assessed most frequently amongst the experiments (32.30%, k = 167). The combination of behavior and physiology (22.82%, k = 118) was most common in experiments assessing two additional parameters, while behavior was the most occurring single domain (13.93%, k = 72).

### 3.5. Outcomes related to study hypothesis

We were interested in the distribution of interventions and whether the experimental outcome aligned with the *a-priori* research hypotheses stated by the respective study authors (Table 2). The hypotheses were classified based on the expected direction of impact of the experimental intervention on animal welfare as stated by the authors of the studies (increasing or diminishing welfare compared to control/baseline).

**Table 2:** Categorization of the proportion and total numbers of experiments (517 experiments in 509 studies) of the research hypothesis (A, B, C) stated by the respective authors of the reviewed studies reporting an effect on GC levels and/or the three additional assessment parameter, behavior, morphology and physiology.

**Hypothesis**	**GC**	**Behavior**	**Morphology**	**Physiology**
**A. Enhancing welfare (51.06%, k** **=** **264)**				
Homogenous effect	38.64%	23.48%	19.70%	15.15%
	k = 102	k = 62	k = 52	k = 40
Heterogenous effect	20.08%	48.48%	18.18%	33.71%
	k = 53	k = 128	k = 48	k = 89
No effect	41.29%	11.74%	20.83%	22.35%
	k = 109	k = 31	k = 55	k = 59
Not measured	N/A	16.29%	41.29%	28.79%
		k = 63	k = 109	k = 76
**B. Diminishing welfare (28.63%, k** **=** **148)**				
Homogenous effect	45.95%	20.95%	15.54%	19.59%
	k = 68	k = 31	k = 23	k = 29
Heterogenous effect	19.59%	42.57%	16.22%	38.51%
	k = 29	k = 63	k = 48	k = 57
No effect	34.46%	12.16%	22.97%	22.30%
	k = 51	k = 18	k = 55	k = 33
Not measured	N/A	24.32%	45.27%	19.59%
		k = 36	k = 109	k = 29
**C. Neutral/no directional hypothesis (20.31%, k** **=** **105)**				
Homogenous effect	25.71%	17.14%	7.62%	8.57%
	k = 27%	k = 18	k = 8	k = 9
Heterogenous effect	33.33%	50.48%	24.76%	40.00%
	k = 35	k = 53	k = 26	k = 42
No effect	40.95%	16.19%	28.57%	19.05%
	k = 43	k = 17	k = 30	k = 20
Not measured	N/A	16.19%	39.05%	32.38%
		k = 17	k = 41	k = 34

Effects are categorized according to the definitions of homogenous (consistent change in readout parameters compared to baseline/control), heterogenous (inconsistent change in readout parameters compared to baseline/control), or no effect (see Table 1). Results are presented in proportions [%] and total number of experiments [k]. The outcomes of the assessment parameters add up to 100% per hypothesis.

Most of the experiments investigated a putative welfare improving intervention, followed by a putative welfare diminishing interventions. The smallest subset of experiments did not formulate a a-priory hypothesis.

If the intervention was expected to improve welfare, most of the experiments found no effect on GC levels, followed by homogenous changes in GC levels. If the intervention was expected to diminish welfare, most of the experiments found a homogenous effect on GC levels, followed by no effect. If authors did not formulate a directional hypothesis, most of the experiments found no effect on GC levels, followed by heterogenous effects. Outcomes in the domain behavior were most often heterogenous (mean 47.18%), irrespective of the hypotheses. Morphological parameters were most frequently not measured (mean 41.87%), or were found not affected by the intervention (mean 24.12%). Effects on physiology were most often heterogenous (mean 37.41%), again, irrespective of the original research hypotheses.

### 3.6. Glucocorticoid sampling

**Figure 3:**
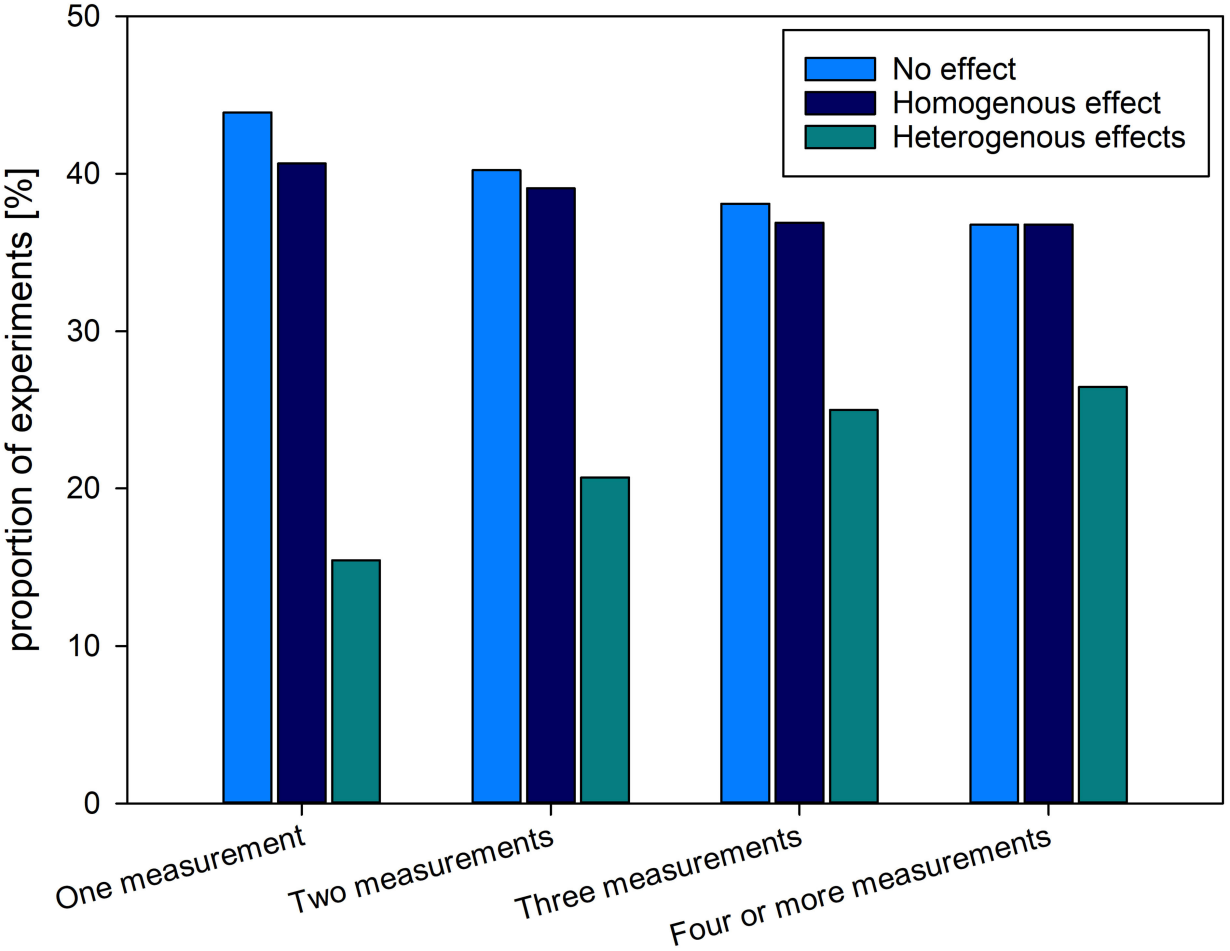
Proportion and total numbers of experiments (517 experiments in 509 studies) finding a homogenous (consistent change in readout parameter compared to baseline/control), heterogenous (inconsistent change in readout parameter compared to baseline/control) or no effect of the intervention on GC levels among the different experimental designs with regard to the number of timepoints at which GC was measured during the experiment. Results are presented in proportions [(%), left vertical axis] and total number of experiments [(k), right vertical axis].

The proportion of homogenous, heterogeneous or no effects on GCs, depending on the number of sampling timepoints is depicted in Figure 3. GCs were measured four and more times (43.13%, k = 223), followed by experiments measuring GC two (16.83%, k = 87), and three times (16.25%, k = 84). GCs were measured only once in roughly one quarter of the experiments (23.79%, k = 123).

With an increasing number of sampling timepoints throughout the experimental period, the proportion of experiments finding no effect on GC levels declined, similarly to experiments finding a homogenous effect. However, the percentage of experiments finding heterogenous effects on GC levels rose with number of GC measurements.

Samples for GC determination were most frequently collected under undisturbed circumstances (aiming at determining baseline concentrations, 78.92%, k = 408). In only 14.70% of the experiments (k = 76), GCs were measured after applying a stressor or challenge (e.g., ACTH), or a combination of both (6.38%, k = 33). If a stressor was applied, 32.89% (k = 25) of the studies found a homogenous effect on GCs, whereas 46.05% (k = 35) did not find an effect. In case there was no stressor applied, 40.44% of the experiments (k = 165) found a homogenous effect on GC, whereas 38.48% (k = 157) did not find an effect.

Regarding the sample matrix and prominent GC metabolite, GC levels were predominantly determined from blood samples, in which mainly cortisol was quantified (Table 3). Less-invasive sampling techniques, such as sampling feces or saliva, ranked second and third, respectively. Table 3 also shows the outcome categories in relation to the sample matrices. Of the experiments that sampled blood and saliva, 40.20% (k = 123) and 45.28% (k = 24), respectively, found homogenous effects, while of the experiments that quantified GCs in feces or urine, 58.33% (k = 49) and 42.86% (k = 9) found no effect.

**Table 3:** Ranking of the proportion and total numbers of experiments (517 experiments in 509 studies) of biological sample types used for determination of GC levels, the corresponding most commonly determined GC metabolite and the outcome of the GC assessment.

**Sample**	**Proportion of total studies**	**GC metabolite (most frequently measured in the corresponding sample)**	**GC outcome (based on all samples)**		
			**Homogenous effect**	**Heterogenous effect**	**No effect**
Blood	59.19% (k = 306)	Cortisol (70.26%, k = 215)	40.20% (k = 123)	23.20% (k = 71)	36.60% (k = 112)
Feces	16.25% (k = 84)	Corticosterone (51.19%, k = 43)	29.76% (k = 25)	11.90% (k = 10)	58.33% (k = 49)
Saliva	10.25% (k = 53)	Cortisol (100.00%, k = 53)	45.28% (k = 24)	22.64% (k = 12)	32.08% (k = 17)
Multiple sample types	6.19% (k = 32)	Cortisol (56.25%, k = 18)	31.25% (k = 10)	40.63% (k = 13)	28.13% (k = 9)
Urine	4.06% (k = 21)	CORT: Creatinine ratio (66.67%, k = 14) [71.43% cortisol (k = 10), 28.57% corticosterone (k = 4)]	38.10% (k = 8)	19.05% (k = 4)	42.86% (k = 9)
Other	2.32% (k = 12)	Cortisol [66.67%, k = 8; whole (fish) body 87.5% (k = 7), milk 12.5% (k = 1)]; Corticosterone [33.33%, k = 4; eggs (avian) 50.00% (k = 2), water (fish) 50.00% (k = 2)]	33.33% (k = 4)	41.67% (k = 5)	25.00% (k = 3)
Hair	1.16% (k = 6)	Cortisol (100.00%, k = 6)	50.00% (k = 3)	50.00% (k = 3)	–
Feathers	0.58% (k = 3)	Corticosterone (100.00%, k = 3)	–	66.67% (k = 2)	33.33% (k = 1)

Note that the most frequently reported GC metabolite is probably confounded by the species most commonly sampled (e.g., cortisol in blood from mammals). Results are given in proportions [%] and total numbers [k] in brackets.

### 3.7. Experimental design

Most experiments, compared treatment to control groups (56.87%, k = 294). Of these experiments, 37.07% (k = 109) found a homogenous effect on GC levels, whereas 40.48% (k = 119) found no effect. A within-subject design was used by 16.44% (k = 85) of the experiments, and 49.25% (k = 33) found a homogenous effect, whereas 32.84% (k = 22) did not find an effect. The combination of between and within subject design was used by 26.69% (k = 138) experiments, of which 3.48% (k = 18) used a cross-over design.

Duration of the intervention was categorized as days to weeks for almost two thirds of the experiments (58.61%, k = 303), followed by months to years (16.83%, k = 87). A proportion of 11.61% (k = 60) of the experiments investigated acute treatments (lasting minutes to hours), 5.03% (k = 26) investigated permanent treatments (from birth till sampling/euthanasia), followed by experimental treatments that were repeatedly continuing (days to weeks; 4.06%, k = 21), repeatedly acute (minutes to hours; 3.29%, k = 17) or repeatedly long-term (months to years; 0.58%, k = 3). Within the category of acute interventions, 46.47% (k = 28) of experiments found homogenous effects on GC responses, in 26.67% (k = 16) no effect was reported. Of the experiments investigating interventions that lasted from days to week, 37.95% (k = 115) found homogenous effects on GC, and 37.62% (k = 114) reported no effects on GC levels. Homogenous effects on GC levels were found in 39.08% (k = 34) of the experiments that investigated long-term interventions, whereas 41.38% (k = 36) found no effect.

**Figure 4:**
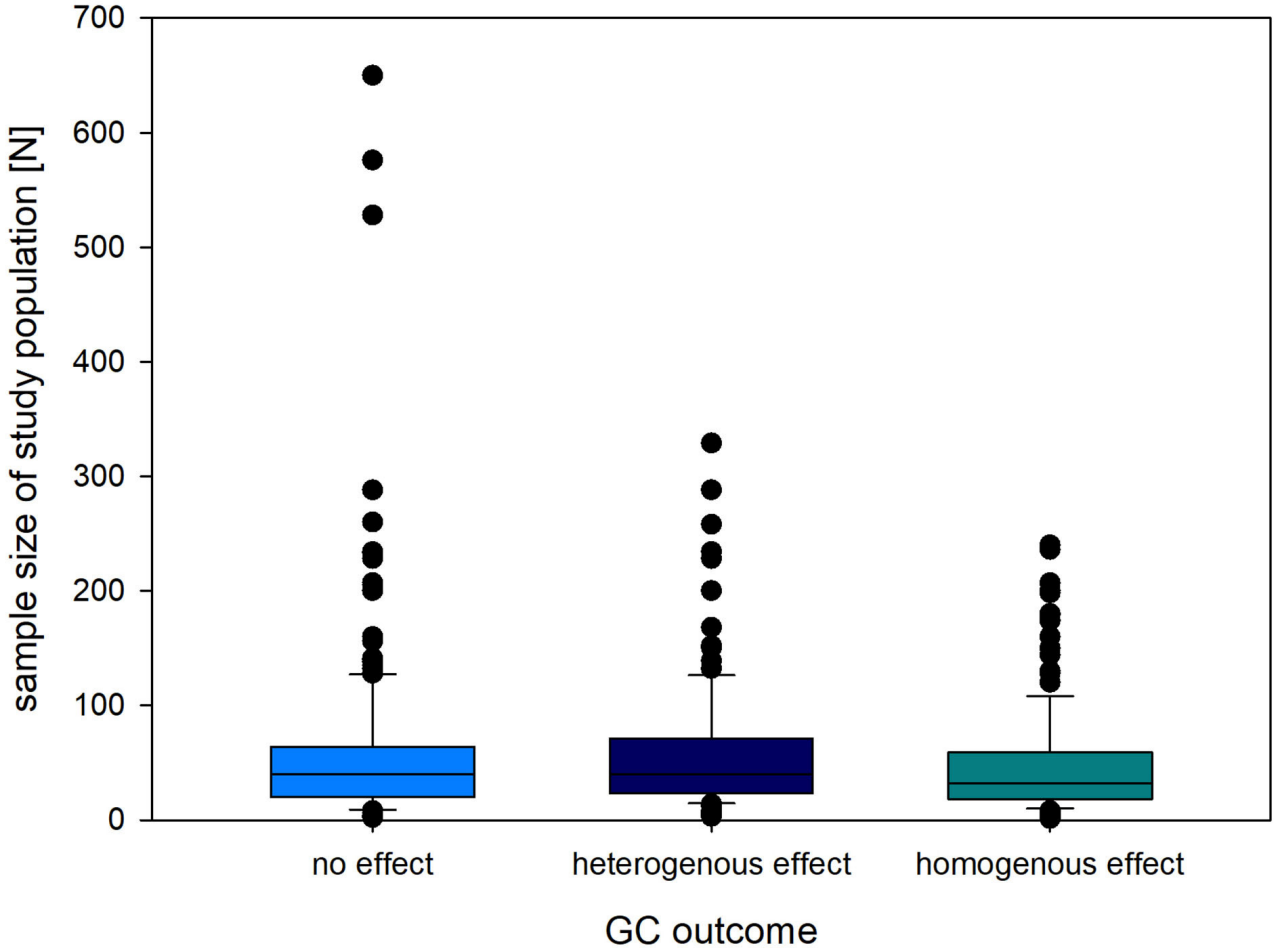
Sample size (*N*) of animals sampled for GCs in studies finding a homogenous (consistent change in readout parameter compared to baseline/control), heterogenous (inconsistent change in readout parameter compared to baseline/control) or no effect of the intervention on GC levels. The box plots represent the median (and 10th, 25th, 75th and 90th percentile) as well as outliers (outside percentiles).

Sample size of the experimental animals used in the reviewed experiments ranged from 1 (orangutan) to 650 (tilapia). In all three GC outcome categories, the median sample size ranged from 32 to 40 individual animals (no effect, homogenous, heterogenous, Figure 4). We further summarized the sample size per class of animals in Table 4.

**Table 4:** Categorization of the proportion and total numbers of experiments (517 experiments in 509 studies) of animal class, occurrence, primary GC sample type, primary additional assessment parameters (B, Behavior; M, Morphology; P, Physiology), primary investigated sex, and average sample size tested for GC levels (*N*).

**Class**	**Proportion of total studies**	**GC sample type**	**Primary additional parameter(s)**	**Primary investigated sex**	**Average sample size**
Mammalian	76.02% (k = 393)	Blood (53.94%, k = 212)	M & B & P (31.81%, k = 125)	Female (35.88%, k = 141)	49 [1; 329]
Avian	14.70% (k = 76)	Blood (76.32%, k = 58)	M & B & P (46.05%, k = 35)	Female (56.58%, k = 43)	74 [6; 576]
Fish	7.93% (k = 41)	Blood (82.93%, k = 34)	M & P (46.34%, k = 19)	Not reported (60.98%, k = 25); both (26.83%, k = 11)	84 [12; 650]
Reptile	0.77% (k = 4)	Blood and Feces (50% each, k = 2)	M & B & P (50.00%, k = 2)	Both (75.00%, k = 3)	16 [3;49]
Amphibian	0.58% (k = 3)	Water extraction (66.67%, k = 2)	Physiology (66.67%, k = 2)	Both (66.67%, k = 2)	47 [15;90]

Results are given in proportions [%] and total numbers [k] in brackets. Average sample size is given in mean numbers of animals [min; max].

### 3.8. Study population

**Figure 5:**
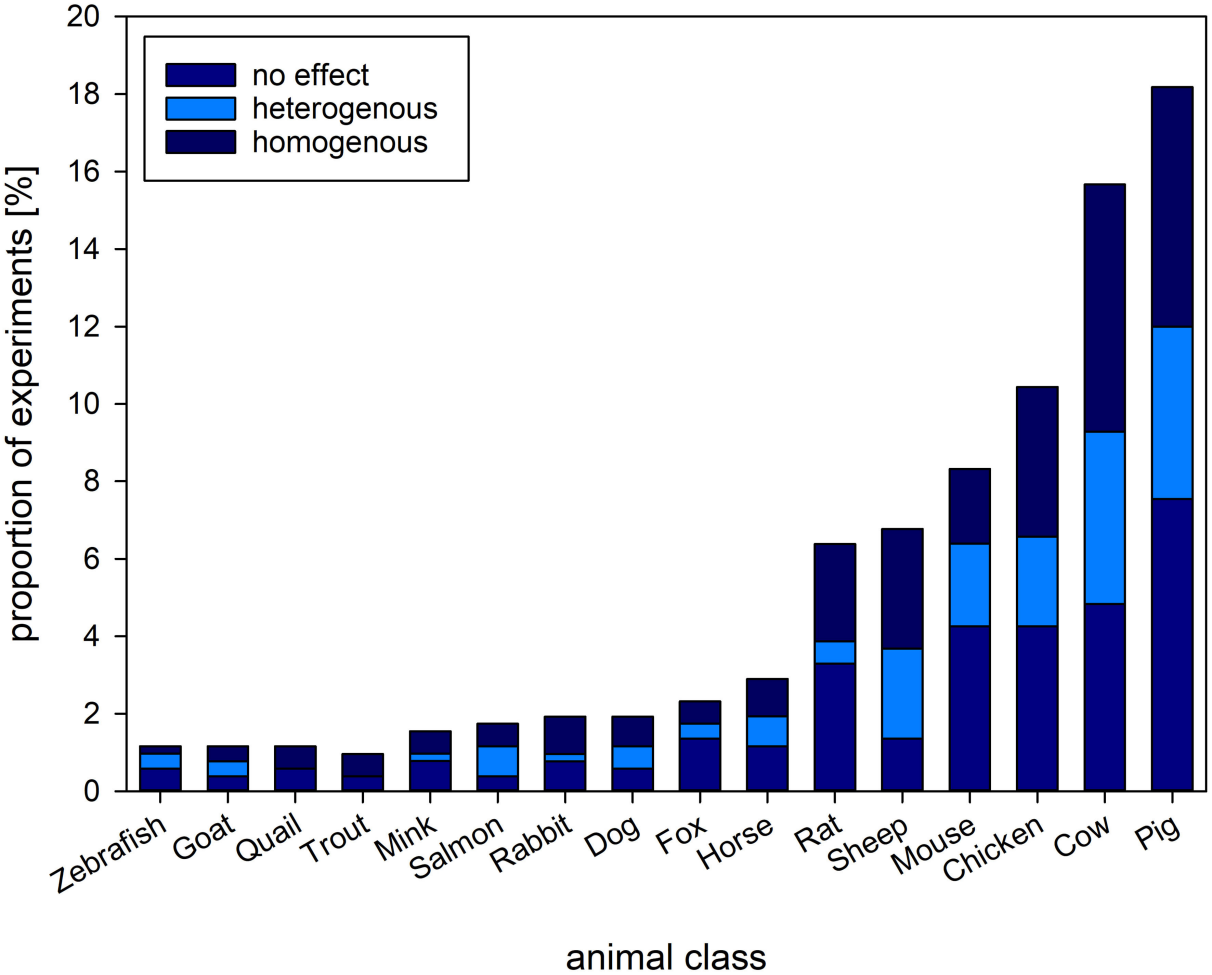
Proportion [%] of experiments and the types of animals investigated. Stacked bars refer to the proportion of experiments reporting on homogenous (consistent change in readout parameter compared to baseline/control), heterogenous (inconsistent change in readout parameter compared to baseline/control) or no effect of an intervention on GC levels found among the species investigated. The figure shows the types of animals with a proportion above 1% of the complete set of experiments.

The reviewed experiments comprised a wide variety of animal species, ranging from zebrafish to elephants. Most of the experiments investigated the welfare of pigs, cows, chicken and sheep (i.e., farm animals, 51.06%, k = 264), or mice and rats (i.e., laboratory animals, 14.70%, k = 76, Figure 5).

We combined the information given for the animal class, the prevalence among experiments, the welfare parameters assessed as well as sex of the animals investigated and sample size of the experiments. To identify over- or under representation of animal classes in welfare research, we combined species to classes to assess their proportion in the reviewed experiments. As welfare assessment may differ between species and classes, we also related the additional assessment parameters to the respective class (Table 4). Three quarters of the selected studies investigated mammals. Blood as GC sampling matrix was most commonly used across animal classes, as also was the combinatory assessment of behavioral, morphological, and physiological parameters. Females were the most often investigated sex in mammals and avian species, whereas in fish studies, sexes were most often not reported. The mean sample size ranged from 16 individuals in reptiles to 84 in fish, with a minimum of 1 in mammals and 650 in avian species.

### 3.9. Interventions

**Figure 6:**
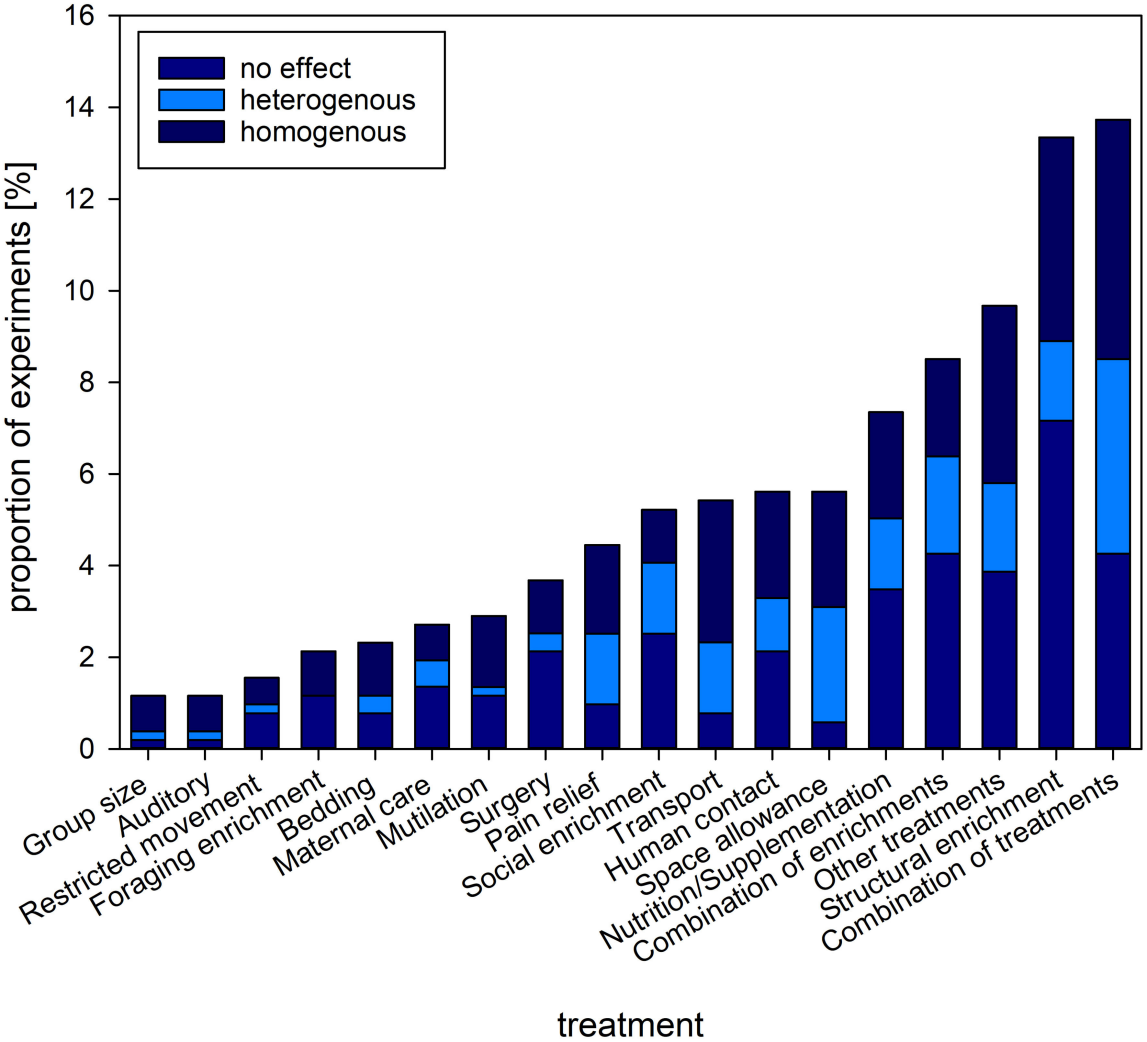
Proportion [%] of experiments and the type of interventions investigated. Stacked bars refer to the proportion of experiments reporting on homogenous (consistent change in readout parameter compared to baseline/control), heterogenous (inconsistent change in readout parameter compared to baseline/control) or no effect of the intervention on GC levels. The figure shows the types of interventions with a proportion above 1% of the complete set of experiments.

Among the interventions aimed at affecting welfare, most of the experiments used a combination of interventions, followed by structural enrichments, other treatments (e.g., milking procedures in dairy cows being the most commonly applied single treatment), combinations of enrichments, and nutrition/nutritional supplementation (Figure 6).

## 4. Discussion

The question leading to this mapping review was whether GCs change consistently in response to a welfare intervention, and whether changes were also reflected in measures of behavior, morphology and physiology. With help of a systematic mapping review of the relevant literature, we examined 509 studies including 517 independent experiments. The included experiments used measurements of GCs in combination with measurements of behavior, morphology, and physiology, to assess the welfare of non-human vertebrates in response to an intervention expected to affect animal welfare. We were specifically interested in the added value of GCs as a proxy indicator in animal welfare studies. While this mapping review started with a comprehensive search and screening, the search and screening were restricted to studies with a focus on welfare. Relevant information from publications with another focus was thus not included, as we were specifically interested in the added value of GCs as a proxy indicator in animal welfare studies.

For a mapping review, the goal is not to perform in-depth (meta) analyses, but rather to describe the available literature, in this case on the use of GCs in the assessment of animal welfare. We present an overview on aspects of experimental set-ups and sampling regimes, the sample types analyzed for quantification of GCs, and the species and sex of the target animals. To identify global patterns in the outcome of GC monitoring as proxy for animal welfare, the interventions and original authors' hypotheses were categorized according to the effects of the interventions on GC levels. Finally, we were interested in whether a significant change in GCs was accompanied by changes in behavioral, morphologic, and physiological parameters.

### 4.1. Network visualization

The network visualization reveals the diversity of welfare-related topics in which GCs are assessed. The clusters formed in the network analyses match current societal and scientific welfare concerns across animals in different contexts such as farm, laboratory and zoological exhibitions (43, 44). Examples are husbandry (light and density), procedures of commercial livestock management (castration), health issues of farm animals (lameness), and the aim to assess emotional states (fear). The selection criteria thus resulted in a representative coverage of the relevant literature.

### 4.2. Effects on glucocorticoids

The results suggest that the prevalence of effects on GC driven by welfare-related interventions is ambiguous. To make informed conclusions, and avoid vote counting, however, the experimental outcomes need to be analyzed on a more detailed level, ideally using meta-analysis approach. Almost two thirds of the reviewed experiments found an effect of the experimental intervention on GC levels, even if heterogenous effects, thus not all measurements showing significant effects, or inconsistent effects. The probability for an intervention to result in heterogenous effects within a domain may rise with the number of readout parameters. For GCs, however, the spread across the outcome categories was similar across number of sampling moments. Note that to statistically confirm these patterns, further detailed analyses are necessary.

The large number of records and variation in experimental set-ups and analyses prevented a clear distinction of outcomes into increase or decrease of GCs with respect to control/baseline. Notably, rather than the direction of change of GC levels, change itself, may be an indicator of an animal being in a state of arousal (45).

Even if GC levels do not change in response to an intervention, this does not mean that modulation of HPA axis and stress responsivity has not taken place. Under some circumstances, changes in patterns of GC release may only be visible when accounting for circadian rhythms [e.g., (46, 47)]. We included only studies which followed the animals longer than 24 h. Short-term GC responses may point to the animal experiencing acute stress, however, to identify and impact on welfare and the individual's capacity to cope and adapt, an animal needs to be followed over a longer period of time (6, 48).

Animals may also develop a hypo- or hyperresponsiveness to stressors, after being exposed to prolonged periods of chronic or repeated stressors (49–51). This modulation of GC baseline and peak levels in response to chronic exposure to stressors may hamper the interpretation of GC responses to interventions, as individuals in the stressor group may have lower baseline GC levels compared to controls (52). The animal may have adapted to the challenge in order to cope by a new physiological setpoint [allostatic state, (48, 53)]. The crucial point is whether adjusted setpoints have negative consequences on the physical and emotional wellbeing of the individual, or on the animal's capacity to reach a state that it perceives as positive.

Also, persisting changes in GC levels can be, but are not necessarily accompanied by structural changes in organs, e.g., changes in the morphology of the adrenal glands (54). Even more variation is added by the sample type reflecting free or bound fractions of GCs or even GC metabolites (30). These may differ between species (29, 55) and need to be identified prior to measuring and interpreting concentrations with respect to the effects of an intervention (56). We show variation in GC metabolites per sample matrix. This pattern may be influenced by the sample matrices that are typically used withing a particular animal class. However, whether the probability to find an effect of a welfare intervention depends on the sample matrix, remains to be investigated. The lack of validation of GC (metabolite) quantification in alternative sample types such as feces, for example, may lead to erroneous results (30).

Other influential factors in the interpretation of GC levels are corticosteroid binding globulins (CBGs), which regulate bioavailability of GCs. The measurement and correction for CBG levels is not yet widely being applied in research on stress and animal welfare, though recognized by critical reviews on the usefulness of GCs for welfare assessment (13–15). Thus, basing conclusions concerning the effect of a welfare related intervention solely on an effect found on the animal's GC levels appears unsubstantiated by the literature. Regarding the determination of GCs, conceptual, and technical challenges need to be met to create standardized and robust data on GC levels, and to aid the interpretation of changes in GC patterns.

### 4.3. Additional assessment parameters

GC levels alone are of limited informational value without additional information about the animal, at least about its current behavior, morphology, and physiology, ideally accompanied by information on ontogeny and previous experiences. The value of GCs may be adding information to other welfare proxies while the correlation between different welfare readout parameters needs further study.

Although the measurement of GCs is often considered to represent valid “welfare measurements,” GCs alone do not seem to be valid indicators of welfare, and additional (or even more suitable) readout parameters need to be identified and used. As we limited our review to studies about welfare assessment, we only included studies in our mapping review that measured additional morphological, behavioral, and/or physiological parameters.

The combination of sampling of GC and collection of parameters from the three domains, behavior, morphology, and physiology, was applied most frequently. This reflects the general consensus that welfare comprises several domains (8). Behavior was the most often assessed additional parameter. This might be due to the unanimous opinion that behavior is the main non-invasive readout parameter to assess the welfare state of an animal, across species and between breeds (57–59). How an individual valences its own emotional state cannot be assessed by other non-invasive procedures than behavioral observations (60). Promising indicators for assessing pain and distress in animals, for example, have been obtained through the analyses of vocalizations (61, 62) and by analyzing the animal's facial expressions, using grimace scales. The latter have successfully been developed for several species (63).

When behavior was assessed as additional parameter to GC values, studies reported heterogenous results. This finding indicates that, if there was an effect of the intervention, measurements of different behavioral traits did not show consistent changes. We did not extract information on type and details of behavioral observations, but the high variability in outcomes urges for adaptation, standardization and validation of tests, a common definition of test aims (i.e., which domain is tested in the animal, e.g., exploration, fear, habituation), and detailed description of test setups.

While we acknowledge behavior as the most important indicator of an individual's emotional state, the use of parameters related to health, morphology, and physiology may be indispensable for a comprehensive approach to welfare. The measurement of physiological parameters is subjected to the same issues as GC measurements, thus variability due to sampling regimen or due to individual phenotypes. Moreover, there is a great variety of parameters to choose from, and therefore it was not surprising that studies reported no or heterogenous effects. The large variation in parameters (and outcomes) calls for detailed reviews identifying overarching patterns in effects on specific readout parameters. While there are many promising physiological parameters to monitor and assess welfare, these also need a thorough validation and should be evaluated in a more in-depth review [e.g., (64, 65)].

Morphological parameters are thought to be closely linked to animal welfare, since physical health is often considered a prerequisite for welfare. In our data set, however, the majority of experiments did not monitor parameters related to morphology. Notably, an animal in good health is not automatically in a state of positive welfare, and vice versa. As long as the individual has not reached the limits of its adaptive capacity, and the health status does not prevent it from reaching a state that it perceives as positive, compromised health may not lead to seriously compromised welfare (66). Monitoring clinical signs and body mass is common practice to assess welfare, as these traits are relatively easy to assess. Monitoring weight loss has been shown to be indicative of severe suffering in laboratory animals, but assessing (milder forms of) distress requires the measurement of additional parameters (67). Therefore, also the use of body mass as primary welfare indicator for at least laboratory animals should be questioned [e.g., (68)]. Regarding animals selected for high productivity, the relation between body mass and welfare may be distorted, e.g., in broiler chicken or pigs, where fear of humans, indicative of a negative welfare state, and productivity are inversely correlated (69, 70).

### 4.4. Outcomes related to study hypothesis

A considerable number of experiments yielded results that were inconsistent with the hypotheses (i.e., no effect while enhancing/diminishing welfare was hypothesized). While this finding can be explained by the ambiguous effects that welfare related interventions may have on assessment parameters, it also highlights the importance of formulating *a-priori*, testable, and clear research hypotheses. Hypothesis-driven studies are especially needed in the field of stress and welfare research, which is complex and faced with subjective attitudes and interpretations (71). Also, the underlying conceptual approach to welfare, and a hypothesis about why GC levels should in-/decrease in response to a certain intervention, should be explicitly stated. Interventions aimed at affecting welfare and not resulting in changes in GCs may support the critical literature on the validity of GCs for the assessment of welfare (13–16). Alternatively, the interventions may have been unsuccessful in affecting welfare. Teasing apart these two aspects requires a critical evaluation of the design of interventions, and its appropriateness for the study population.

It is a common misperception that GC levels equal the levels of stress an animal experiences (12), and that stress equals diminished welfare (72). We argue that an animal resides in a positive welfare state as long as it can cope and adapt to the demands of its (prevailing) environmental circumstances, enabling it to reach a state that it perceives as positive, e.g., that evokes positive emotions (3, 6). Thus, when investigating the effects of stressors, these aspects should be considered when formulating the research hypothesis.

Since we extracted the hypotheses as they were presented in the final publications, our results may have been affected by authors adapting their hypotheses in the writing phase to improve storytelling. Recent work shows that this phenomenon, generally referred to as “HARKing” (Hypothesizing After Results are Known) also exists in animal welfare research (19). The high incidence of inconsistency between the hypotheses and results reported in the set of reviewed studies suggests that HARKing in the welfare field is less common. Alternatively, the proportion of inconclusive results may even be larger than can be deduced from the published records, i.e., the proportion of inconsistent results may be an underrepresentation of the real number of studies that did not support the (original) hypothesis. This idea may become testable as soon as a priori protocol registration becomes common practice and original hypotheses can reliably be retrieved.

#### 4.4.1. Glucocorticoid sampling

To elucidate potential improvements regarding the informative value of GCs as welfare proxy, we extracted information on sampling methods. Most often, experiments sampled GCs more than once, in line with our conceptual approach to welfare, which is dynamic (3, 6). The proportion of experiments finding homogenous effects or no effects were comparable across the number of GC measurements, but the proportion of experiments reporting heterogenous effects increased. Note that long-term experiments, in which the GC levels initially changed due to the intervention and then returned to control or baseline levels, were not scored as heterogenous but included in the homogenous category because this change was considered as temporary.

To identify patterns of GC release in response to an acute stressor, multiple measurements on the dynamics of the GC response may prove to be more informative about the animal's coping style and resilience (73). Further long-term and in-depth analyses are needed to understand the time course of HPA axis regulation, the role of allostasis and resilience, and the consequences for welfare (74).

Blood sampling for cortisol was the most common procedure. Animal species differ in the glucocorticoid that is predominantly produced by the adrenals, e.g., corticosterone in birds and rodents, and cortisol in most other mammals. However, research has shown that local GC and GC metabolite synthesis may lead to local differences in which glucocorticoid is present at a higher level, challenging the idea that species are corticosterone or cortisol dominant [e.g., (75)]. In addition, cortisol and corticosterone concentrations might not correlate (76). Especially studies investigating the immune system as index of welfare should consider measuring several GC metabolites when trying to elucidate mechanisms [e.g., (77)].

Nearly half of the experiments collected sample matrices such as feces, urine, hair, or other substances (e.g., milk or eggs) to quantify GC levels. These matrices allow for less invasive sampling than blood collection. Additionally, the procedures necessary to collect blood samples may affect levels of GCs themselves (78). Therefore, alternative sample matrices are promising for repeatedly sampling to avoid accumulation of discomfort. Notably, handling to collect alternative samples, e.g., placing the animal in a separate cage [e.g., (79)], may affect GC levels in subsequent samples. Moreover, the relation between GC levels in blood and in alternative matrices needs more investigation. Sample matrices reflect different time periods of GC accumulation (56), thus relative levels might not relate between sample types (23). Interestingly, minimally invasively measured levels of GCs, e.g., in feces or in feathers, have been shown to reflect biologically meaningful patterns (80, 81). Further validation of measurements, e.g., by challenging animals with ACTH and subsequently collecting samples over a period of time to determine peak GC concentrations, is highly recommended (29, 30).

Next to handling of the animals, several technical challenges may account, at least partly, for the variation in the relation between GCs and additional proxies of welfare. Sampling regimens should account for variation on the individual instead of group/cage level (82–84). A circadian and circannual rhythm [e.g., (85)] may lead to additional variation, which needs to be controlled experimentally and statistically. The subsequent processing of samples in the laboratory—i.e., whether and how the sample is extracted and purified, the choice of antibody and assay type—may further impact measured concentrations (86), though a recent meta-analysis did not find an effect of assay method (87).

### 4.5. Experimental design

A between-subject design was applied in most of the reviewed experiments, comparing the effects of a treatment with a control group. Regarding to the importance of the individual in welfare concepts, data on pre-intervention values would add valuable information about individual profiles (88). Vice versa, studies investigating within-individual changes may miss the comparison to a control group over time. One third of the reviewed studies did indeed use a combined experimental set-up, thus included within- and between individual, or group, measurements. Long-term effects of an intervention may then become visible, which is important in view of the concept that welfare is dynamic and dependent on the individuals' adaptive capacity.

All three GC outcome domains were prevalent across a range of sample sizes, but detailed analyses are needed to infer the likelihood of finding effects on GC levels are driven by the number of animals sampled. Information on sample size for GC measurements proved rather difficult to retrieve. We therefore urge the reporting of precise sample sizes along with the statistics or graphical representations of GC levels. A sufficiently large sample size and appropriate statistical power is an issue across research topics. Guidelines for animal welfare research have been published to ensure the appropriate samples size in animal welfare studies, especially when investigating adverse interventions (89).

#### 4.5.1. Study population

Most of the experiments focused on farm animals such as pigs, cows, and chicken, and on laboratory animals such as mice and rats, followed by other domesticated species. These results may indicate the great effort of researchers investigating welfare and welfare-related interventions in the two most frequently used animal clusters (44). The proportion of studies aiming to study the welfare of a given species might also be linked to the perceived ethical conflict regarding the intrinsic value of animals, urging to safeguard the animal's integrity on the one side, and the use of animals for human purposes, on the other (90). The perceived need for improvement of husbandry circumstances seems to be highest in pigs as well as other species used for meat production.

Despite the increasing production of fish in commercial aquaculture, studies investigating a welfare related intervention in fish were scarce in our data set. The small number of studies represented in our review may be due to our selection criteria, as research on fish welfare often assesses measurements directly relating to the stress response and does not include additional parameter such as behavior. Given the large numbers of fish used in intensive aquaculture, and the large variety of fish species used, more research on their biological needs and potential welfare, using appropriate readout parameter is clearly needed (91, 92). We therefore advocate investigating the biological needs and to identify readout parameters suitable for assessing welfare in species underrepresented in our data set, such as amphibians, reptiles, and fish (93–96).

### 4.6. Interventions

Prior to data extraction, the team of reviewers defined a set of categories of interventions, based on our knowledge of the field. We were able to assign the major part of the experiments to these categories, however, ten percent were defined as “other intervention.” The outcome confirms the broad scope of welfare related research, and the complexity of factors to which the animals are exposed to. The clusters found are similar to the clusters of the network analysis from Freire and Nicol (44).

The variation of interventions, even within categories, may partly explain the variation in outcome of welfare proxies such as GCs, and behavior, morphology, and physiology. It remains to be statistically evaluated whether certain interventions lead to specific patterns in GC responses. Moreover, the type and array of welfare assessment parameters should be adjusted to the type of intervention.

Structural enrichment was the main category of single interventions, followed by a combination of enrichments. Enrichment aims to trigger different motivational systems and stimulates the animal to express its full behavioral repertoire (97). Similarly, space allowance and restricted movement were often investigated. Both determine which behaviors an animal can express, as some systems severely restrict movement (e.g., gestation stalls in sows). Expressing natural and/or normal behavior is recognized as a biological need and crucial for the animal to reach a positive welfare state (98, 99). Assessment of welfare based on GCs and other physiological parameters may not be appropriate in these systems, as physical exercise may affect the outcomes, leading to e.g., higher GC levels (100).

### 4.7. Limitations

This mapping review provides an overview of experiments systematically in- or excluded according to a list of a priori criteria. While some of the exclusion criteria were based on a clear go/no go decision, others may be debatable. The first exclusion criterium, invertebrate, or human study population, served to limit the review to animal species in which GCs are components of the stress response. Based on the recent insights into emotional capacities in invertebrate species, we urge to extend welfare concepts and assessment protocols to these taxa (101).

Given the variety of opinions on welfare, the decision whether an experiment was assessing welfare or purely focused on the physiological response to stressors was challenging. Based on the conceptual approach that welfare is dynamic and comprises several domains (4), we established the criterium that, next to GCs, additional parameters needed to be measured (out of the domains, behavior, morphology and physiology) and that the experiment needed to follow the animals longer than 24 h. The 24 h cut-off was chosen to distinguish the acute GC stress response from long-term consequences affecting the coping capacity of animals (32). Nevertheless, we claim that 24 h are certainly not long enough to truly reflect coping capacity and welfare. Welfare assessment of an individual should take its lifetime experiences into account, positive as well as negative, as reflected by concepts such as “a Life Worth Living” (102, 103). Important for the welfare of an individual is that also acute interventions may have long-term effects, especially if applied during sensitive phases such as early life or adolescence, or even prenatally (104–106). If we, however, would have selected only studies across a lifetime of an animal, we would have significantly limited our sample size, as permanent interventions represented only a very small proportion of our set of publications. Nevertheless, our dataset offers the future possibility to investigate the included studies in more detail.

We did not extract detailed quantitative information about GC levels or the other outcome parameter. Instead, we chose to provide an overview of studies measuring GCs as part of assessing animal welfare. We did not analyze relations between experimental characteristics and outcome parameter to prevent “vote counting” (40). Vote counting, thus adding up the number of studies finding an effect, while omitting the statistical analysis of these studies, may lead to a false interpretation and over- or underestimation of effect sizes. While summations may suggest an effect of an intervention (or the absence thereof), reliable interpretation is only possible by performing a weighted meta-analysis on the summarized data, taking means and variation into account (40). Keeping this in mind, we describe GC, behavioral, morphological, and physiological outcomes to provide an overview of the field, not inferring statistical implications. The here-presented results therefore do not allow for causal inferences, rather serves as inspiration. Further exploration of the data set, focusing on a carefully selected and defined subset of limited criteria and parameters, offers the opportunity to perform a meta-analysis and support further results by applying appropriate statistical analyses.

## 5. Conclusions and recommendations

The variation in outcomes of GCs, behavioral, morphological, and physiological parameter indicates that we have not yet developed the toolbox to decide with sufficient certainly whether an intervention improves or compromises welfare. Further research relating these parameters to the emotional experience of an individual, e.g., in terms of positive welfare indicators, is crucial (107). The complexity and multidimensional nature of welfare assessment needs, and benefits from, more than one assessment dimension (108).

This mapping review provides one of the largest explorative analyses of experimental studies using GCs as one of the parameters to assess the effects of interventions on animal welfare. The results are meant to encourage further extraction and quantitative meta-analysis of the relationships between parameter, for example testing effects on GCs in relation to intervention or sample matrix. Further research aiding our understanding of the interplay of GCs and behavioral, morphological and physiological processes, is clearly needed.

## Data availability statement

The data that support the findings of this study are available from the corresponding author, IT upon reasonable request: inga.tiemann@uni-bonn.de.

## Author contributions

VG and IT conceived the presented idea. IT, LF, MB, CL, and VG defined the literature search. IT, LF, MB, EL, and VG carried out the literature search. IT and VG wrote the paper with input from all the co-authors. All authors contributed to the article and approved the submitted version.

## References

[1] Welfare Quality^®^. Welfare Quality^®^ Assessment Protocol for Cattle. Lelystad: Welfare Quality^®^ (2009).

[2] Brambell FW Barbour DS Lady Barnett Ewer TK Hobson A Pitchforth H . Report of the Technical Committee to Enquire Into the Welfare of Animals Kept Under Intensive Livestock Husbandry Systems. London (1965).

[3] Ohl F van der Staay FJ. Animal welfare: at the interface between science and society. Vet J. (2012) 192:13–9. 10.1016/j.tvjl.2011.05.019 21703888

[4] Mellor DJ Beausoleil NJ Littlewood KE McLean AN McGreevy PD Jones B . The 2020 five domains model: including human–animal interactions in assessments of animal welfare. Animals. (2020) 10:1870. 10.3390/ani10101870 33066335 PMC7602120

[5] Mellor DJ Reid C. Concepts of animal well-being and predicting the impact of procedures on experimental animals. In: Improving the Well-Being of Animals in the Research Environment. Glen Osmond, SA: WellBeing International (1994). p. 3–18. Available online at: https://www.wellbeingintlstudiesrepository.org/exprawel/7/

[6] Arndt SS Goerlich VC van der Staay FJ. A dynamic concept of animal welfare: the role of appetitive and adverse internal and external factors and the animal's ability to adapt to them. Front Anim Sci. (2022) 3:908513. 10.3389/fanim.2022.908513

[7] Boissy A Manteuffel G Bak Jensen M Oppermann Moe R Spruijt B Keeling LJ . Assessment of positive emotions in animals to improve their welfare. Physiol Behav. (2007) 92:375–97. 10.1016/j.physbeh.2007.02.003 17428510

[8] Mellor DJ. Operational details of the five domains model and its key applications to the assessment and management of animal welfare. Animals. (2017) 7:60. 10.3390/ani7080060 28792485 PMC5575572

[9] Rushen J. Using aversion learning techniques to assess the mental state, suffering, and welfare of farm animals. J Anim Sci. (1996) 74:1990–5. 10.2527/1996.7481990x 8856455

[10] Mason G Mendl M. Why is there no simple way of measuring animal welfare? Anim. Welf. (1993) 2:301–19.

[11] Farm Animal Welfare Council. Farm Animal Welfare in Great Britain: Past, Present and Future. London (2009).

[12] MacDougall-Shackleton SA Bonier F Romero LM Moore IT. Glucocorticoids and “stress” are not synonymous. Integrat Organ Biol. (2019) 1:obz017. 10.1093/iob/obz017 33791532 PMC7671118

[13] Otovic P Hutchinson E. Limits to using HPA axis activity as an indication of animal welfare. ALTEX Alter Anim Exp. (2015) 32:41–50. 10.14573/altex.1406161 25418851

[14] Mormède P Andanson S Aupérin B Beerda B Guémené D Malmkvist J . Exploration of the hypothalamic–pituitary–adrenal function as a tool to evaluate animal welfare. Physiol Behav. (2007) 92:317–39. 10.1016/j.physbeh.2006.12.003 17234221

[15] Ralph CR Tilbrook AJ. The usefulness of measuring glucocorticoids for assessing animal welfare. J Anim Sci. (2016) 94:457–70. 10.2527/jas.2015-9645 27065116

[16] Rushen J. Problems associated with the interpretation of physiological data in the assessment of animal welfare. Appl Anim Behav Sci. (1991) 28:381–6. 10.1016/0168-1591(91)90170-3

[17] Wiepkema PR Koolhaas JM. Stress and animal welfare. Anim Welf. (1993) 2:195–218.

[18] Veissier I Boissy A. Stress and welfare: two complementary concepts that are intrinsically related to the animal's point of view. Physiol Behav. (2007) 92:429–33. 10.1016/j.physbeh.2006.11.008 17182067

[19] van der Schot AA Phillips C. Publication bias in animal welfare scientific literature. J Agric Environ Ethics. (2013) 26:945–58. 10.1007/s10806-012-9433-8

[20] Harris BN Carr JA. The role of the hypothalamus-pituitary-adrenal/interrenal axis in mediating predator-avoidance trade-offs. Gen Comp Endocrinol. (2016) 230:110–42. 10.1016/j.ygcen.2016.04.006 27080550

[21] Sapolsky RM Romero LM Munck AU. How do glucocorticoids influence stress responses? Integrating permissive, suppressive, stimulatory, and preparative actions. Endocrine Rev. (2000) 21:55–89. 10.1210/edrv.21.1.0389 10696570

[22] Jimeno B Hau M Verhulst S. Corticosterone levels reflect variation in metabolic rate, independent of ‘stress'. Sci Rep. (2018) 8:13020. 10.1038/s41598-018-31258-z 30158537 PMC6115469

[23] Cook NJ. Review: minimally invasive sampling media and the measurement of corticosteroids as biomarkers of stress in animals. Can J Anim Sci. (2012) 92:227–59. 10.4141/cjas2012-045

[24] Borg KE Esbenshade KL Johnson BH. Cortisol, growth hormone, and testosterone concentrations during mating behavior in the bull and boar. J Anim Sci. (1991) 69:3230–40. 10.2527/1991.6983230x 1894559

[25] Buwalda B Scholte J Boer SF de Coppens CM Koolhaas JM. The acute glucocorticoid stress response does not differentiate between rewarding and aversive social stimuli in rats. Horm Behav. (2012) 61:218–26. 10.1016/j.yhbeh.2011.12.012 22210197

[26] Fairhurst GD Frey MD Reichert JF Szelest I Kelly DM Bortolotti GR. Does environmental enrichment reduce stress? An integrated measure of corticosterone from feathers provides a novel perspective. PLoS ONE. (2011) 6:e17663. 10.1371/journal.pone.0017663 21412426 PMC3055884

[27] Bienertova-Vasku J Lenart P Scheringer M. Eustress and distress: neither good nor bad, but rather the same? BioEssays. (2020) 42:1900238. 10.1002/bies.201900238 32302008

[28] Meehan CL Mench JA. The challenge of challenge: can problem solving opportunities enhance animal welfare? Appl Anim Behav Sci. (2007) 102:246–61. 10.1016/j.applanim.2006.05.031

[29] Touma C Palme R. Measuring fecal glucocorticoid metabolites in mammals and birds: the importance of validation. Ann N Y Acad Sci. (2005) 1046:54–74. 10.1196/annals.1343.006 16055843

[30] Palme R. Non-invasive measurement of glucocorticoids: advances and problems. Physiol Behav. (2019) 199:229–43. 10.1016/j.physbeh.2018.11.021 30468744

[31] Spencer RL Deak T. A users guide to HPA axis research. Physiol Behav. (2017) 178:43–65. 10.1016/j.physbeh.2016.11.014 27871862 PMC5451309

[32] García A Martí O Vallès A Dal-Zotto S Armario A. Recovery of the hypothalamic-pituitary-adrenal response to stress. NEN. (2000) 72:114–25. 10.1159/000054578 10971146

[33] Leenaars CH van der Mierden S Durst M Goerlich-Jansson VC Ripoli FL Keubler LM . Measurement of corticosterone in mice: a protocol for a mapping review. Lab Anim. (2020) 54:26–32. 10.1177/0023677219868499 31657274

[34] Cockrem JF. Individual variation in glucocorticoid stress responses in animals. Gen Comp Endocrinol. (2013) 181:45–58. 10.1016/j.ygcen.2012.11.025 23298571

[35] Eriksen MB Frandsen TF. The impact of patient, intervention, comparison, outcome (PICO) as a search strategy tool on literature search quality: a systematic review. JMLA. (2018) 106:420–31. 10.5195/jmla.2018.345 30271283 PMC6148624

[36] Leenaars M Hooijmans CR van Veggel N Riet G ter Leeflang M Hooft L . A step-by-step guide to systematically identify all relevant animal studies. Lab Anim. (2012) 46:24–31. 10.1258/la.2011.011087 22037056 PMC3265183

[37] de Vries R Hooijmans CR Tillema A Leenaars M Ritskes-Hoitinga M. Updated version of the Embase search filter for animal studies. Lab Anim. (2014) 48:88. 10.1177/0023677213494374 23836850

[38] Ouzzani M Hammady H Fedorowicz Z Elmagarmid A. Rayyan—a web and mobile app for systematic reviews. Syst Rev. (2016) 5:210. 10.1186/s13643-016-0384-4 27919275 PMC5139140

[39] van der Mierden S Tsaioun K Bleich A Leenaars CH. Software tools for literature screening in systematic reviews in biomedical research. ALTEX Alter Anim Exp. (2019) 36:508–17. 10.14573/altex.1902131 31113000

[40] Borenstein M Hedges LV Higgins JP Rothstein HR. Introduction to Meta-Analysis. Oxford: John Wiley and Sons (2021).

[41] Waltman L van Eck NJ Noyons EC. A unified approach to mapping and clustering of bibliometric networks. J Informetr. (2010) 4:629–35. 10.1016/j.joi.2010.07.002

[42] Page MJ McKenzie JE Bossuyt PM Boutron I Hoffmann TC Mulrow CD . The PRISMA 2020 statement: an updated guideline for reporting systematic reviews. BMJ. (2021) 372:n71. 10.1136/bmj.n71 33782057 PMC8005924

[43] Walker M Diez-Leon M Mason G. Animal welfare science: recent publication trends and future research priorities. Int J Consum Stud. (2014) 27:80–100. 10.46867/ijcp.2014.27.01.03

[44] Freire R Nicol CJ. A bibliometric analysis of past and emergent trends in animal welfare science. Anim Welf. (2019) 28:465–85. 10.7120/09627286.28.4.465

[45] Dickens MJ Romero LM. A consensus endocrine profile for chronically stressed wild animals does not exist. Gen Comp Endocrinol. (2013) 191:177–89. 10.1016/j.ygcen.2013.06.014 23816765

[46] de Jong IC Prelle I Vandeburgwal J Lambooij E Korte S Blokhuis H . Effects of environmental enrichment on behavioral responses to novelty, learning, and memory, and the circadian rhythm in cortisol in growing pigs. Physiol Behav. (2000) 68:571–8. 10.1016/S0031-9384(99)00212-7 10713299

[47] Naderi F Hernández-Pérez J Chivite M Soengas JL Míguez JM López-Patiño MA. Involvement of cortisol and sirtuin1 during the response to stress of hypothalamic circadian system and food intake-related peptides in rainbow trout, oncorhynchus mykiss. Chronobiol Int. (2018) 35:1122–41. 10.1080/07420528.2018.1461110 29737878

[48] Korte SM Olivier B Koolhaas JM. A new animal welfare concept based on allostasis. Physiol Behav. (2007) 92:422–8. 10.1016/j.physbeh.2006.10.018 17174361

[49] Cyr NE Michael Romero L. Chronic stress in free-living European starlings reduces corticosterone concentrations and reproductive success. Gen Comp Endocrinol. (2007) 151:82–9. 10.1016/j.ygcen.2006.12.003 17280663

[50] Rich EL Romero LM. Exposure to chronic stress downregulates corticosterone responses to acute stressors. Am J Physiol Regul Integrat Comp Physiol. (2005) 288:R1628–36. 10.1152/ajpregu.00484.2004 15886358

[51] Wulsin AC Wick-Carlson D Packard BA Morano R Herman JP. Adolescent chronic stress causes hypothalamo–pituitary–adrenocortical hypo-responsiveness and depression-like behavior in adult female rats. Psychoneuroendocrinology. (2016) 65:109–17. 10.1016/j.psyneuen.2015.12.004 26751968 PMC4968078

[52] Romero LM Beattie UK. Common myths of glucocorticoid function in ecology and conservation. J Exp Zool Part A Ecol Integrat Physiol. (2022) 337:7–14. 10.1002/jez.2459 33819389

[53] McEwen BS. Stress, adaptation, and disease: allostasis and allostatic load. Ann N Y Acad Sci. (1998) 840:33–44. 10.1111/j.1749-6632.1998.tb09546.x 9629234

[54] Kasanen IH Inhilä KJ Vainio OM Kiviniemi VV Hau J Scheinin M . The diet board: welfare impacts of a novel method of dietary restriction in laboratory rats. Lab Anim. (2009) 43:215–23. 10.1258/la.2008.008066 19237451

[55] Gayrard V Alvinerie M Toutain PL. Interspecies variations of corticosteroid-binding globulin parameters. Domest Anim Endocrinol. (1996) 13:35–45. 10.1016/0739-7240(95)00042-9 8625614

[56] Gormally BM Romero LM Angelier F. What are you actually measuring? A review of techniques that integrate the stress response on distinct time-scales. Funct Ecol. (2020) 34:2030–44. 10.1111/1365-2435.13648

[57] Dawkins MS. Using behaviour to assess animal welfare. Anim Welf. (2004) 13:3–7.

[58] Mason GJ Mench J. Using behaviour to assess animal welfare. In: Animal Welfare. Wallingford: CAB International (2021). p. 127–41.

[59] Meuser V Weinhold L Hillemacher S Tiemann I. Welfare-related behaviors in chickens: characterization of fear and exploration in local and commercial chicken strains. Animals. (2021) 11:679. 10.3390/ani11030679 33806293 PMC7998364

[60] Mendl M Burman OH Paul ES. An integrative and functional framework for the study of animal emotion and mood. Proc R Soc B Biol Sci. (2010) 277:2895–904. 10.1098/rspb.2010.0303 20685706 PMC2982018

[61] Manteuffel G Puppe B Schön PC. Vocalization of farm animals as a measure of welfare. Appl Anim Behav Sci. (2004) 88:163–82. 10.1016/j.applanim.2004.02.012

[62] Mcloughlin MP Stewart R McElligott AG. Automated bioacoustics: methods in ecology and conservation and their potential for animal welfare monitoring. J R Soc Interf. (2019) 16:20190225. 10.1098/rsif.2019.0225 31213168 PMC6597774

[63] Mogil JS Pang DS Silva Dutra GG Chambers CT. The development and use of facial grimace scales for pain measurement in animals. Neurosci Biobehav Rev. (2020) 116:480–93. 10.1016/j.neubiorev.2020.07.013 32682741

[64] von Borell E Langbein J Després G Hansen S Leterrier C Marchant-Forde J . Heart rate variability as a measure of autonomic regulation of cardiac activity for assessing stress and welfare in farm animals — a review. Physiol Behav. (2007) 92:293–316. 10.1016/j.physbeh.2007.01.007 17320122

[65] Jerez-Cepa I Ruiz-Jarabo I. Physiology: an important tool to assess the welfare of aquatic animals. Biology. (2021) 10:61. 10.3390/biology10010061 33467525 PMC7830356

[66] Duncan IJ Petherick JC. The implications of cognitive processes for animal welfare. J Anim Sci. (1991) 69:5017–22. 10.2527/1991.69125017x 1808195

[67] Talbot SR Biernot S Bleich A van Dijk RM Ernst L Häger C . Defining body-weight reduction as a humane endpoint: a critical appraisal. Lab Anim. (2020) 54:99–110. 10.1177/0023677219883319 31665969

[68] Kalliokoski O Jacobsen KR Darusman HS Henriksen T Weimann A Poulsen HE . Mice do not habituate to metabolism cage housing–a three week study of male BALB/c mice. PLoS ONE. (2013) 8:e58460. 10.1371/journal.pone.0058460 23505511 PMC3591308

[69] Hemsworth PH Barnett JL Hansen C. The influence of handling by humans on the behavior, growth, and corticosteroids in the juvenile female pig. Horm Behav. (1981) 15:396–403. 10.1016/0018-506X(81)90004-0 7327535

[70] Hemsworth PH Coleman GJ Barnett JL Jones RB. Behavioural responses to humans and the productivity of commercial broiler chickens. Appl Anim Behav Sci. (1994) 41:101–14. 10.1016/0168-1591(94)90055-8 10700627

[71] Harris BN. Stress hypothesis overload: 131 hypotheses exploring the role of stress in tradeoffs, transitions, and health. Gen Comp Endocrinol. (2020) 288:113355. 10.1016/j.ygcen.2019.113355 31830473

[72] Tilbrook AJ Ralph CR. Hormones, stress and the welfare of animals. Anim Product Sci. (2018) 58:408. 10.1071/AN16808

[73] Ericsson M Fallahsharoudi A Bergquist J Kushnir MM Jensen P. Domestication effects on behavioural and hormonal responses to acute stress in chickens. Physiol Behav. (2014) 133:161–9. 10.1016/j.physbeh.2014.05.024 24878317

[74] Nazar FN Estevez I. The immune-neuroendocrine system, a key aspect of poultry welfare and resilience. Poult Sci. (2022) 101:101919. 10.1016/j.psj.2022.101919 35704954 PMC9201016

[75] Schmidt KL Soma KK. Cortisol and corticosterone in the songbird immune and nervous systems: local vs. systemic levels during development. Am J Physiol Regulat Integrat Comp Physiol. (2008) 295:R103–R110. 10.1152/ajpregu.00002.2008 18353885

[76] Koren L Whiteside D Fahlman Å Ruckstuhl K Kutz S Checkley S . Cortisol and corticosterone independence in cortisol-dominant wildlife. Gen Comp Endocrinol. (2012) 177:113–9. 10.1016/j.ygcen.2012.02.020 22449618

[77] Tetel V Tonissen S Fraley GS. Sex difference in changes in heterophil to lymphocyte ratios in response to acute exposure of both corticosterone and cortisol in the Pekin duck. Poult Sci. (2022) 101:101914. 10.1016/j.psj.2022.101914 35551001 PMC9108750

[78] Newman AE Hess H Woodworth BK Norris DR. Time as tyrant: the minute, hour and day make a difference for corticosterone concentrations in wild nestlings. Gen Comp Endocrinol. (2017) 250:80–4. 10.1016/j.ygcen.2017.05.022 28577897

[79] Nicholson A Malcolm RD Russ PL Cough K Touma C Palme R . The response of C57BL/6J and BALB/cJ mice to increased housing density. J Am Assoc Lab Anim Sci. (2009) 48:14. 19930822 PMC2786928

[80] Lind M-A Hõrak P Sepp T Meitern R. Corticosterone levels correlate in wild-grown and lab-grown feathers in greenfinches (*Carduelis chloris*) and predict behaviour and survival in captivity. Horm Behav. (2020) 118:104642. 10.1016/j.yhbeh.2019.104642 31765655

[81] Sheriff MJ Krebs CJ Boonstra R. Assessing stress in animal populations: do fecal and plasma glucocorticoids tell the same story? Gen Comp Endocrinol. (2010) 166:614–9. 10.1016/j.ygcen.2009.12.017 20051245

[82] Alm M Wall H Holm L Wichman A Palme R Tauson R. Welfare and performance in layers following temporary exclusion from the litter area on introduction to the layer facility. Poult Sci. (2015) 94:565–73. 10.3382/ps/pev021 25681475

[83] Gjendal K Sørensen DB Kiersgaard MK Ottesen JL. Hang on: an evaluation of the hemp rope as environmental enrichment in C57BL/6 mice. Anim. Welf. (2017) 26:437–47. 10.7120/09627286.26.4.437

[84] Ronca AE Moyer EL Talyansky Y Lowe M Padmanabhan S Choi S . Behavior of mice aboard the international space station. Sci Rep. (2019) 9:4717. 10.1038/s41598-019-40789-y 30976012 PMC6459880

[85] Rao R Androulakis IP. The physiological significance of the circadian dynamics of the HPA axis: interplay between circadian rhythms, allostasis and stress resilience. Horm Behav. (2019) 110:77–89. 10.1016/j.yhbeh.2019.02.018 30862458

[86] Beyl HE Jimeno B Lynn SE Breuner CW. Assay temperature affects corticosteroid-binding globulin and free corticosterone estimates across species. Gen Comp Endocrinol. (2021) 310:113810. 10.1016/j.ygcen.2021.113810 33964285

[87] van der Mierden S Leenaars CH Boyle EC Ripoli FL Gass P Durst M . Measuring endogenous corticosterone in laboratory mice - a mapping review, meta-analysis, and open source database. ALTEX Alter Anim Exp. (2021) 38:111–22. 10.14573/altex.2004221 33086382

[88] van der Goot MH Boleij H van den Broek J Salomons AR Arndt SS van Lith HA. An individual based, multidimensional approach to identify emotional reactivity profiles in inbred mice. J Neurosci Methods. (2020) 343:108810. 10.1016/j.jneumeth.2020.108810 32574640

[89] Hampton JO MacKenzie DI Forsyth DM. How many to sample? Statistical guidelines for monitoring animal welfare outcomes. PLoS ONE. (2019) 14:e0211417. 10.1371/journal.pone.0211417 30699193 PMC6353194

[90] Röcklinsberg H Gamborg C Gjerris M. A case for integrity: gains from including more than animal welfare in animal ethics committee deliberations. Lab Anim. (2014) 48:61–71. 10.1177/0023677213514220 24367033

[91] Franks B Ewell C Jacquet J. Animal welfare risks of global aquaculture. Sci Adv. (2021) 7:eabg0677. 10.1126/sciadv.abg0677 33811081 PMC11057778

[92] Toni M Manciocco A Angiulli E Alleva E Cioni C Malavasi S. Review: assessing fish welfare in research and aquaculture, with a focus on European directives. Animal. (2019) 13:161–70. 10.1017/S1751731118000940 29717679

[93] Colson V Mure A Valotaire C Le Calvez JM Goardon L Labbé L . A novel emotional and cognitive approach to welfare phenotyping in rainbow trout exposed to poor water quality. Appl Anim Behav Sci. (2019) 210:103–12. 10.1016/j.applanim.2018.10.010

[94] Michaels CJ Downie JR Campbell-Palmer R. The importance of enrichment for advancing amphibian welfare and conservation goals: a review of a neglected topic. Biodiversity. (2014) 8:17.

[95] Pasmans F Bogaerts S Braeckman J Cunningham AA Hellebuyck T Griffiths RA . Future of keeping pet reptiles and amphibians: towards integrating animal welfare, human health and environmental sustainability. Vet Rec. (2017) 181:450. 10.1136/vr.104296 29051315

[96] Silla AJ Calatayud NE Trudeau VL. Amphibian reproductive technologies: approaches and welfare considerations. Conserv Physiol. (2021) 9:coab011. 10.1093/conphys/coab011 33763231 PMC7976225

[97] Newberry RC. Environmental enrichment: increasing the biological relevance of captive environments. Appl Anim Behav Sci. (1995) 44:229–43. 10.1016/0168-1591(95)00616-Z

[98] Bracke MB Hopster H. Assessing the importance of natural behavior for animal welfare. J Agric Environ Ethics. (2006) 19:77–89. 10.1007/s10806-005-4493-7

[99] Špinka M. How important is natural behaviour in animal farming systems? Appl Anim Behav Sci. (2006) 100:117–28. 10.1016/j.applanim.2006.04.006

[100] Veissier I Andanson S Dubroeucq H Pomiès D. The motivation of cows to walk as thwarted by tethering. J Anim Sci. (2008) 86:2723–9. 10.2527/jas.2008-1020 18539832

[101] Horvath K Angeletti D Nascetti G Carere C. Invertebrate welfare: an overlooked issue. Ann Ist Super Sanita. (2013) 49:9–17. 10.4415/ANN_13_01_04 23535125

[102] Mellor DJ. Updating animal welfare thinking: moving beyond the “five freedoms” towards “a life worth living”. Animals. (2016) 6:21. 10.3390/ani6030021 27102171 PMC4810049

[103] Yeates JW. Is 'a life worth living' a concept worth having? Anim Welf. (2011) 20:397–406.

[104] Begni V Sanson A Pfeiffer N Brandwein C Inta D Talbot SR . Social isolation in rats: effects on animal welfare and molecular markers for neuroplasticity. PLoS ONE. (2020) 15:e0240439. 10.1371/journal.pone.0240439 33108362 PMC7591026

[105] Costa JH Cantor MC Adderley NA Neave HW. Key animal welfare issues in commercially raised dairy calves: social environment, nutrition, and painful procedures. Can J Anim Sci. (2019) 99:649–60. 10.1139/cjas-2019-0031

[106] Rutherford K Donald R Arnott G Rooke J Dixon L Mehers JJ . Farm animal welfare: assessing risks attributable to the prenatal environment. Anim Welf. (2012) 21:419–29. 10.7120/09627286.21.3.419

[107] Kremer L Klein Holkenborg SEJ Reimert I Bolhuis JE Webb LE. The nuts and bolts of animal emotion. Neurosci Biobehav Rev. (2020) 113:273–86. 10.1016/j.neubiorev.2020.01.028 31982603

[108] Botreau R Veissier I Butterworth A Bracke MB Keeling LJ. Definition of criteria for overall assessment of animal welfare. Anim Welf. (2007) 16:225–8.

