# Supplementary material for: Glucocorticoids in relation to behavior, morphology, and physiology as proxy indicators for the assessment of animal welfare. A systematic mapping review
Source: Front Vet Sci. 2023 Jan 6;9:954607. doi: 10.3389/fvets.2022.954607 (PMC9853183; doi:10.3389/fvets.2022.954607)
Supplement: Supplementary file 1 [file Table_1.DOCX]

***Supplementary Material***

# **Glucocorticoids and Animal Welfare Assessment**

# Search string GCs and Animal Welfare Assessment

## 1. PUBMED

### SC1 Animal Welfare

(animal welfare [Mesh] OR animal welfare [tiab] OR (animal [tiab] AND welfare [tiab]) OR animal wellbeing [tiab] OR (animal [tiab] AND wellbeing [tiab]) OR animal well-being [tiab] OR (animal [tiab] AND well-being [tiab]))

### SC2 Glucocorticoids

(corticosterone[mesh] OR corticosterone[tiab] OR corticocorticosterone [tiab] OR corticocosteroid[tiab] OR corticorterone[tiab] OR corticoserone[tiab] OR corticostcrone[tiab] OR corticosteone[tiab] OR corticoster[tiab] OR corticosterene[tiab] OR corticostereone[tiab] OR corticosteron[tiab] OR cortikosterone[tiab] OR cortikosteron[tiab] OR kortikosterone[tiab] OR kortikosteron[tiab] OR ‘kendall compound b’[tiab] OR ‘reichstein substance h’[tiab] OR corticoesterone[tiab] OR cortecosterone[tiab] OR corticossterone[tiab] OR cortcosterone[tiab] OR cortisol[tiab] OR cortisol levels[tiab] OR cortisone[tiab] OR cortizone[tiab] OR corticosteroid[tiab] OR corticosteroids[tiab] OR ‘adrenal cortical steroid’[tiab] OR corticoid[tiab] OR corticoids[tiab] OR cortef[tiab] OR hydrocortone[tiab] OR hydrocortisone[tiab] OR glucocorticoids[tiab] OR glucocorticoid[tiab] OR glucocorticosteroid[tiab] OR glucocorticosteroids[tiab])

### SC3 All Non-human animals excluding invertebrates

((“animal experimentation”[MeSH Terms] OR “models, animal”[MeSH Terms] OR “Animals”[Mesh:noexp] OR “animal population groups”[MeSH Terms] OR “chordata”[MeSH Terms:noexp] OR “vertebrates”[MeSH Terms:noexp] OR “amphibians”[MeSH Terms] OR “birds”[MeSH Terms] OR “fishes”[MeSH Terms] OR “reptiles”[MeSH Terms] OR “mammals”[MeSH Terms:noexp] OR “primates”[MeSH Terms:noexp] OR “artiodactyla”[MeSH Terms] OR “carnivora”[MeSH Terms] OR “cetacea”[MeSH Terms] OR “chiroptera”[MeSH Terms] OR “elephants”[MeSH Terms] OR “hyraxes”[MeSH Terms] OR “insectivora”[MeSH Terms] OR “lagomorpha”[MeSH Terms] OR “marsupialia”[MeSH Terms] OR “monotremata”[MeSH Terms] OR “perissodactyla”[MeSH Terms] OR “rodentia”[MeSH Terms] OR “scandentia”[MeSH Terms] OR “sirenia”[MeSH Terms] OR “xenarthra”[MeSH Terms] OR “haplorhini”[MeSH Terms:noexp] OR “strepsirhini”[MeSH Terms] OR “platyrrhini”[MeSH Terms] OR “tarsii”[MeSH Terms] OR “catarrhini”[MeSH Terms:noexp] OR “cercopithecidae”[MeSH Terms] OR “hylobatidae”[MeSH Terms] OR “hominidae”[MeSH Terms:noexp] OR “gorilla gorilla”[MeSH Terms] OR “pan paniscus”[MeSH Terms] OR “pan troglodytes”[MeSH Terms] OR “pongo pygmaeus”[MeSH Terms]) OR ((animals[tiab] OR animal[tiab] OR pet[tiab] OR pets[tiab] OR mice[tiab] OR mus[tiab] OR mouse[tiab] OR murine[tiab] OR woodmouse[tiab] OR rats[tiab] OR rat[tiab] OR murinae[tiab] OR muridae[tiab] OR cottonrat[tiab] OR cottonrats[tiab] OR hamster[tiab] OR hamsters[tiab] OR cricetinae[tiab] OR rodentia[tiab] OR rodent[tiab] OR rodents[tiab] OR pigs[tiab] OR pig[tiab] OR swine[tiab] OR swines[tiab] OR piglets[tiab] OR piglet[tiab] OR boar[tiab] OR boars[tiab] OR “sus scrofa”[tiab] OR ferrets[tiab] OR ferret[tiab] OR polecat[tiab] OR polecats[tiab] OR “mustela putorius”[tiab] OR “guinea pigs”[tiab] OR “guinea pig”[tiab] OR cavia[tiab] OR callithrix[tiab] OR marmoset[tiab] OR marmosets[tiab] OR cebuella[tiab] OR hapale[tiab] OR octodon[tiab] OR chinchilla[tiab] OR chinchillas[tiab] OR gerbillinae[tiab] OR gerbil[tiab] OR gerbils[tiab] OR jird[tiab] OR jirds[tiab] OR merione[tiab] OR meriones[tiab] OR rabbits[tiab] OR rabbit[tiab] OR hares[tiab] OR hare[tiab] OR diptera[tiab] OR flies[tiab] OR fly[tiab] OR dipteral[tiab] OR drosophila[tiab] OR drosophilidae[tiab] OR cats[tiab] OR cat[tiab] OR carus[tiab] OR felis[tiab] OR nematoda[tiab] OR nematode[tiab] OR nematodes[tiab] OR sipunculida[tiab] OR dogs[tiab] OR dog[tiab] OR canine[tiab] OR canines[tiab] OR canis[tiab] OR sheep[tiab] OR sheeps[tiab] OR mouflon[tiab] OR mouflons[tiab] OR ovis[tiab] OR goats[tiab] OR goat[tiab] OR capra[tiab] OR capras[tiab] OR rupicapra[tiab] OR rupicapras[tiab] OR chamois[tiab] OR haplorhini[tiab] OR monkey[tiab] OR monkeys[tiab] OR anthropoidea[tiab] OR anthropoids[tiab] OR saguinus[tiab] OR tamarin[tiab] OR tamarins[tiab] OR leontopithecus[tiab] OR hominidae[tiab] OR ape[tiab] OR apes[tiab] OR “pan paniscus”[tiab] OR bonobo[tiab] OR bonobos[tiab] OR “pan troglodytes”[tiab] OR gibbon[tiab] OR gibbons[tiab] OR siamang[tiab] OR siamangs[tiab] OR nomascus[tiab] OR symphalangus[tiab] OR chimpanzee[tiab] OR chimpanzees[tiab] OR prosimian[tiab] OR prosimians[tiab] OR “bush baby”[tiab] OR bush babies[tiab] OR galagos[tiab] OR galago[tiab] OR pongidae[tiab] OR gorilla[tiab] OR gorillas[tiab] OR “pongo pygmaeus”[tiab] OR orangutan[tiab] OR orangutans[tiab] OR lemur[tiab] OR lemurs[tiab] OR lemuridae[tiab] OR horse[tiab] OR horses[tiab] OR equus[tiab] OR cow[tiab] OR calf[tiab] OR bull[tiab] OR avian[tiab] OR avians[tiab] OR chicken[tiab] OR chickens[tiab] OR gallus[tiab] OR quail[tiab] OR duck[tiab] OR goose[tiab] OR geese[tiab] OR turkey[tiab] OR bird[tiab] OR birds[tiab] OR quails[tiab] OR poultry[tiab] OR poultries[tiab] OR fowl[tiab] OR fowls[tiab] OR reptile[tiab] OR reptilia[tiab] OR reptiles[tiab] OR snakes[tiab] OR snake[tiab] OR lizard[tiab] OR lizards[tiab] OR alligator[tiab] OR alligators[tiab] OR crocodile[tiab] OR crocodiles[tiab] OR turtle[tiab] OR turtles[tiab] OR amphibian[tiab] OR amphibians[tiab] OR amphibia[tiab] OR frog[tiab] OR frogs[tiab] OR bombina[tiab] OR salientia[tiab] OR toad[tiab] OR toads[tiab] OR “epidalea calamita”[tiab] OR salamander[tiab] OR salamanders[tiab] OR eel[tiab] OR eels[tiab] OR fish[tiab] OR fishes[tiab] OR pisces[tiab] OR catfish[tiab] OR catfishes[tiab] OR siluriformes[tiab] OR arius[tiab] OR heteropneustes[tiab] OR sheatfish[tiab] OR perch[tiab] OR perches[tiab] OR percidae[tiab] OR perca[tiab] OR trout[tiab] OR trouts[tiab] OR char[tiab] OR chars[tiab] OR salvelinus[tiab] OR minnow[tiab] OR cyprinidae[tiab] OR carps[tiab] OR carp[tiab] OR zebrafish[tiab] OR zebrafishes[tiab] OR goldfish[tiab] OR goldfishes[tiab] OR guppy[tiab] OR guppies[tiab] OR chub[tiab] OR chubs[tiab] OR tinca[tiab] OR barbels[tiab] OR barbus[tiab] OR pimephales[tiab] OR promelas[tiab] OR “poecilia reticulata”[tiab] OR mullet[tiab] OR mullets[tiab] OR eel[tiab] OR eels[tiab] OR seahorse[tiab] OR seahorses[tiab] OR mugil curema[tiab] OR atlantic cod[tiab] OR shark[tiab] OR sharks[tiab] OR catshark[tiab] OR anguilla[tiab] OR salmonid[tiab] OR salmonids[tiab] OR whitefish[tiab] OR whitefishes[tiab] OR salmon[tiab] OR salmons[tiab] OR sole[tiab] OR solea[tiab] OR lamprey[tiab] OR lampreys[tiab] OR pumpkinseed[tiab] OR sunfish[tiab] OR sunfishes[tiab] OR tilapia[tiab] OR tilapias[tiab] OR turbot[tiab] OR turbots[tiab] OR flatfish[tiab] OR flatfishes[tiab] OR sciuridae[tiab] OR squirrel[tiab] OR squirrels[tiab] OR chipmunk[tiab] OR chipmunks[tiab] OR suslik[tiab] OR susliks[tiab] OR vole[tiab] OR voles[tiab] OR lemming[tiab] OR lemmings[tiab] OR muskrat[tiab] OR muskrats[tiab] OR lemmus[tiab] OR otter[tiab] OR otters[tiab] OR marten[tiab] OR martens[tiab] OR martes[tiab] OR weasel[tiab] OR badger[tiab] OR badgers[tiab] OR ermine[tiab] OR mink[tiab] OR minks[tiab] OR sable[tiab] OR sables[tiab] OR gulo[tiab] OR gulos[tiab] OR wolverine[tiab] OR wolverines[tiab] OR mustela[tiab] OR llama[tiab] OR llamas[tiab] OR alpaca[tiab] OR alpacas[tiab] OR camelid[tiab] OR camelids[tiab] OR guanaco[tiab] OR guanacos[tiab] OR chiroptera[tiab] OR chiropteras[tiab] OR bat[tiab] OR bats[tiab] OR fox[tiab] OR foxes[tiab] OR iguana[tiab] OR iguanas[tiab] OR “xenopus laevis”[tiab] OR parakeet[tiab] OR parakeets[tiab] OR parrot[tiab] OR parrots[tiab] OR donkey[tiab] OR donkeys[tiab] OR mule[tiab] OR mules[tiab] OR zebra[tiab] OR zebras[tiab] OR shrew[tiab] OR shrews[tiab] OR bison[tiab] OR bisons[tiab] OR buffalo[tiab] OR buffaloes[tiab] OR deer[tiab] OR deers[tiab] OR bear[tiab] OR bears[tiab] OR panda[tiab] OR pandas[tiab] OR “wild hog”[tiab] OR “wild boar”[tiab] OR fitchew[tiab] OR fitch[tiab] OR beaver[tiab] OR beavers[tiab] OR jerboa[tiab] OR jerboas[tiab] OR capybara[tiab] OR capybaras[tiab] OR canine[tiab] OR bovine[tiab] OR porcine[tiab] OR hog[tiab] OR hogs[tiab]) NOT medline[sb]))

## EMBASE

### SC1 Animal Welfare

‘animal welfare’ OR (‘animal’:ab,kw,ti AND ‘welfare’:ab,kw,ti) OR ‘animal wellbeing’ OR (‘animal’:ab,kw,ti AND ‘wellbeing’:ab,kw,ti) OR ‘animal well-being’ OR (‘animal’:ab,kw,ti AND ‘well-being’:ab,kw,ti)

### SC2 Glucocorticoids

Corticosterone (‘corticosterone’/de OR ‘corticosterone blood level’/de OR ‘corticosterone release’/de OR ‘corticosterone’:ab,kw,ti OR ‘corticocorticosterone’:ab,kw,ti OR ‘corticocosteroid’:ab,kw,ti OR ‘corticorterone’:ab,kw,ti OR ‘corticoserone’:ab,kw,ti OR ‘corticostcrone’:ab,kw,ti OR ‘corticosteone’:ab,kw,ti OR ‘corticoster’:ab,kw,ti OR ‘corticosterene’:ab,kw,ti OR ‘corticostereone’:ab,kw,ti OR ‘corticosteron’:ab,kw,ti OR ‘cortikosterone’:ab,kw,ti OR ‘cortikosteron’:ab,kw,ti OR ‘kortikosterone’:ab,kw,ti OR ‘kortikosteron’:ab,kw,ti OR ‘kendall compound b’:ab,kw,ti OR ‘reichstein substance h’:ab,kw,ti OR ‘cortcosterone’:ab,kw,ti OR ‘corticoesterone’:ab,kw,ti OR ‘corticostron’:ab,kw,ti OR ‘cortocosterone’:ab,kw,ti OR ‘cortisol’:ab,kw,ti OR ‘cortisol levels’:ab,kw,ti OR ‘cortisone’:ab,kw,ti OR ‘cortizone’:ab,kw,ti OR ‘corticosteroid’:ab,kw,ti OR ‘corticosteroids’:ab,kw,ti OR ‘adrenal cortical steroid’:ab,kw,ti OR ‘corticoid’:ab,kw,ti OR ‘corticoids’:ab,kw,ti OR ‘cortef’:ab,kw,ti OR ‘hydrocortone’:ab,kw,ti OR ‘hydrocortisone’:ab,kw,ti OR ‘glucocorticoids’:ab,kw,ti OR ‘glucocorticoid’:ab,kw,ti OR ‘glucocorticosteroid’:ab,kw,ti OR ‘glucocorticosteroids’:ab,kw,ti)

### SC3 All Non-human animals excluding invertebrates

(‘animal experiment’ OR ‘animal model’ OR ‘experimental animal’ OR ‘transgenic animal’ OR ‘male animal’ OR ‘female animal’ OR ‘juvenile animal’ OR ‘animal’ OR ‘chordata’ OR ‘vertebrate’ OR ‘tetrapod’ OR ‘fish’ OR ‘amniote’ OR ‘amphibia’ OR ‘mammal’ OR ‘reptile’ OR ‘sauropsid’ OR ‘therian’ OR ‘monotremate’ OR ‘placental mammals’ OR ’marsupial’ OR ‘Euarchontoglires’ OR ‘Afrotheria’ OR ‘Boreoeutheria’ OR ‘Laurasiatheria’ OR ‘Xenarthra’ OR ‘primate’ OR ‘Dermoptera’ OR ‘Glires’ OR ‘Scandentia’ OR ‘Haplorhini’ OR ‘prosimian’ OR ‘simian’ OR ‘tarsiiform’ OR ‘Catarrhini’ OR ‘Platyrrhini’ OR ‘ape’ OR ‘Cercopithecidae’ OR ‘hominid’ OR ‘hylobatidae’ OR ‘chimpanzee’ OR ‘gorilla’ OR ‘orang utan’) OR (‘animal’ OR ‘animals’ OR ‘pet’ OR ‘pets’ OR ‘pisces’ OR ‘fish’ OR ‘fishes’ OR ‘catfish’ OR ‘catfishes’ OR ‘sheatfish’ OR ‘silurus’ OR ‘arius’ OR ‘heteropneustes’ OR ‘clarias’ OR ‘gariepinus’ OR ‘fathead minnow’ OR ‘fathead minnows’ OR ‘pimephales’ OR ‘promelas’ OR ‘cichlidae’ OR ‘trout’ OR ‘trouts’ OR ‘char’ OR ‘chars’ OR ‘salvelinus’ OR ‘salmo’ OR ‘oncorhynchus’ OR ‘guppy’ OR ‘guppies’ OR ‘millionfish’ OR ‘poecilia’ OR ‘goldfish’ OR ‘goldfishes’ OR ‘carassius’ OR ‘auratus’ OR ‘mullet’ OR ‘mullets’ OR ‘mugil’ OR ‘curema’ OR ‘shark’ OR ‘sharks’ OR ‘cod’ OR ‘cods’ OR ‘gadus’ OR ‘morhua’ OR ‘carp’ OR ‘carps’ OR ‘cyprinus’ OR ‘carpio’ OR ‘killifish’ OR ‘eel’ OR ‘eels’ OR ‘anguilla’ OR ‘zander’ OR ‘sander’ OR ‘lucioperca’ OR ‘stizostedion’ OR ‘turbot’ OR ‘turbots’ OR ‘psetta’ OR ‘flatfish’ OR ‘flatfishes’ OR ‘plaice’ OR ‘pleuronectes’ OR ‘platessa’ OR ‘tilapia’ OR ‘tilapias’ OR ‘oreochromis’ OR ‘sarotherodon’ OR ‘common sole’ OR ‘dover sole’ OR ‘solea’ OR ‘zebrafish’ OR ‘zebrafishes’ OR ‘danio’ OR ‘rerio’ OR ‘seabass’ OR ‘dicentrarchus’ OR ‘labrax’ OR ‘morone’ OR ‘lamprey’ OR ‘lampreys’ OR ‘petromyzon’ OR ‘pumpkinseed’ OR ‘pumpkinseeds’ OR ‘lepomis’ OR ‘gibbosus’ OR ‘herring’ OR ‘clupea’ OR ‘harengus’ OR ‘amphibia’ OR ‘amphibian’ OR ‘amphibians’ OR ‘anura’ OR ‘salientia’ OR ‘frog’ OR ‘frogs’ OR ‘rana’ OR ‘toad’ OR ‘toads’ OR ‘bufo’ OR ‘xenopus’ OR ‘laevis’ OR ‘bombina’ OR ‘epidalea’ OR ‘calamita’ OR ‘salamander’ OR ‘salamanders’ OR ‘newt’ OR ‘newts’ OR ‘triturus’ OR ‘reptilia’ OR ‘reptile’ OR ‘reptiles’ OR ‘bearded dragon’ OR ‘pogona’ OR ‘vitticeps’ OR ‘iguana’ OR ‘iguanas’ OR ‘lizard’ OR ‘lizards’ OR ‘anguis fragilis’ OR ‘turtle’ OR ‘turtles’ OR ‘snakes’ OR ‘snake’ OR ‘aves’ OR ‘bird’ OR ‘birds’ OR ‘quail’ OR ‘quails’ OR ‘coturnix’ OR ‘bobwhite’ OR ‘colinus’ OR ‘virginianus’ OR ‘poultry’ OR ‘poultries’ OR ‘fowl’ OR ‘fowls’ OR ‘chicken’ OR ‘chickens’ OR ‘gallus’ OR ‘zebra finch’ OR ‘taeniopygia’ OR ‘guttata’ OR ‘canary’ OR ‘canaries’ OR ‘serinus’ OR ‘canaria’ OR ‘parakeet’ OR ‘parakeets’ OR ‘grasskeet’ OR ‘parrot’ OR ‘parrots’ OR ‘psittacine’ OR ‘psittacines’ OR ‘shelduck’ OR ‘tadorna’ OR ‘goose’ OR ‘geese’ OR ‘turkey’ OR ‘avian’ OR ‘avians’ OR ‘branta’ OR ‘leucopsis’ OR ‘woodlark’ OR ‘lullula’ OR ‘flycatcher’ OR ‘ficedula’ OR ‘hypoleuca’ OR ‘dove’ OR ‘doves’ OR ‘geopelia’ OR ‘cuneata’ OR ‘duck’ OR ‘ducks’ OR ‘greylag’ OR ‘graylag’ OR ‘anser’ OR ‘harrier’ OR ‘circus pygargus’ OR ‘red knot’ OR ‘great knot’ OR ‘calidris’ OR ‘canutus’ OR ‘godwit’ OR ‘limosa’ OR ‘lapponica’ OR ‘meleagris’ OR ‘gallopavo’ OR ‘jackdaw’ OR ‘corvus’ OR ‘monedula’ OR ‘ruff’ OR ‘philomachus’ OR ‘pugnax’ OR ‘lapwing’ OR ‘peewit’ OR ‘plover’ OR ‘vanellus’ OR ‘swan’ OR ‘cygnus’ OR ‘columbianus’ OR ‘bewickii’ OR ‘gull’ OR ‘chroicocephalus’ OR ‘ridibundus’ OR ‘albifrons’ OR ‘great tit’ OR ‘parus’ OR ‘aythya’ OR ‘fuligula’ OR ‘streptopelia’ OR ‘risoria’ OR ‘spoonbill’ OR ‘platalea’ OR ‘leucorodia’ OR ‘blackbird’ OR ‘turdus’ OR ‘merula’ OR ‘blue tit’ OR ‘cyanistes’ OR ‘pigeon’ OR ‘pigeons’ OR ‘columba’ OR ‘pintail’ OR ‘anas’ OR ‘starling’ OR ‘sturnus’ OR ‘owl’ OR ‘athene noctua’ OR ‘pochard’ OR ‘ferina’ OR ‘cockatiel’ OR ‘nymphicus’ OR ‘hollandicus’ OR ‘skylark’ OR ‘alauda’ OR ‘tern’ OR ‘sterna’ OR ‘teal’ OR ‘crecca’ OR ‘oystercatcher’ OR ‘haematopus’ OR ‘ostralegus’ OR ‘shrew’ OR ‘shrews’ OR ‘sorex’ OR ‘araneus’ OR ‘crocidura’ OR ‘russula’ OR ‘european mole’ OR ‘talpa’ OR ‘chiroptera’ OR ‘bat’ OR ’bats’ OR ‘eptesicus’ OR ‘serotinus’ OR ‘myotis’ OR ‘dasycneme’ OR ‘daubentonii’ OR ‘pipistrelle’ OR ‘pipistrellus’ OR ‘cat’ OR ‘cats’ OR ‘felis’ OR ‘catus’ OR ‘feline’ OR ‘dog’ OR ‘dogs’ OR ‘canis’ OR ‘canine’ OR ‘canines’ OR ‘otter’ OR ‘otters’ OR ‘lutra’ OR ‘badger’ OR ‘badgers’ OR ‘meles’ OR ‘fitchew’ OR ‘fitch’ OR ‘foumart’ OR ‘foulmart’ OR ‘ferrets’ OR ‘ferret’ OR ‘polecat’ OR ‘polecats’ OR ‘mustela’ OR ‘putorius’ OR ‘weasel’ OR ‘weasels’ OR ‘fox’ OR ‘foxes’ OR ‘vulpes’ OR ‘common seal’ OR ‘phoca’ OR ‘vitulina’ OR ‘grey seal’ OR ‘halichoerus’ OR ‘horse’ OR ‘horses’ OR ‘equus’ OR ‘equine’ OR ‘equidae’ OR ‘donkey’ OR ‘donkeys’ OR ‘mule’ OR ‘mules’ OR ‘pig’ OR ‘pigs’ OR ‘swine’ OR ‘swines’ OR ‘hog’ OR ‘hogs’ OR ‘boar’ OR ‘boars’ OR ‘porcine’ OR ‘piglet’ OR ‘piglets’ OR ‘sus’ OR ‘scrofa’ OR ‘llama’ OR ‘llamas’ OR ‘lama’ OR ‘glama’ OR ‘deer’ OR ‘deers’ OR ‘cervus’ OR ‘elaphus’ OR ‘cow’ OR ‘cows’ OR ‘bos taurus’ OR ‘bos indicus’ OR ‘bovine’ OR ‘bull’ OR ‘bulls’ OR ‘cattle’ OR ‘bison’ OR ‘bisons’ OR ‘sheep’ OR ‘sheeps’ OR ‘ovis aries’ OR ‘ovine’ OR ‘lamb’ OR ‘lambs’ OR ‘mouflon’ OR ‘mouflons’ OR ‘goat’ OR ‘goats’ OR ‘capra’ OR ‘caprine’ OR ‘chamois’ OR ‘rupicapra’ OR ‘leporidae’ OR ‘lagomorpha’ OR ‘lagomorph’ OR ‘rabbit’ OR ‘rabbits’ OR ‘oryctolagus’ OR ‘cuniculus’ OR ‘laprine’ OR ‘hares’ OR ‘lepus’ OR ‘rodentia’ OR ‘rodent’ OR ‘rodents’ OR ‘murinae’ OR ‘mouse’ OR ‘mice’ OR ‘mus’ OR ‘musculus’ OR ‘murine’ OR ‘woodmouse’ OR ‘apodemus’ OR ‘rat’ OR ‘rats’ OR ‘rattus’ OR ‘norvegicus’ OR ‘guinea pig’ OR ‘guinea pigs’ OR ‘cavia’ OR ‘porcellus’ OR ‘hamster’ OR ‘hamsters’ OR ‘mesocricetus’ OR ‘cricetulus’ OR ‘cricetus’ OR ‘gerbil’ OR ‘gerbils’ OR ‘jird’ OR ‘jirds’ OR ‘meriones’ OR ‘unguiculatus’ OR ‘jerboa’ OR ‘jerboas’ OR ‘jaculus’ OR ‘chinchilla’ OR ‘chinchillas’ OR ‘beaver’ OR ‘beavers’ OR ‘castor fiber’ OR ‘castor canadensis’ OR ‘sciuridae’ OR ‘squirrel’ OR ‘squirrels’ OR ‘sciurus’ OR ‘chipmunk’ OR ‘chipmunks’ OR ‘marmot’ OR ‘marmots’ OR ‘marmota’ OR ‘suslik’ OR ‘susliks’ OR ‘spermophilus’ OR ‘cynomys’ OR ‘cottonrat’ OR ‘cottonrats’ OR ‘sigmodon’ OR ‘vole’ OR ‘voles’ OR ‘microtus’ OR ‘myodes’ OR ‘glareolus’ OR ‘primate’ OR ‘primates’ OR ‘prosimian’ OR ‘prosimians’ OR ‘lemur’ OR ‘lemurs’ OR ‘lemuridae’ OR ‘loris’ OR ‘bush baby’ OR ‘bush babies’ OR ‘bushbaby’ OR ‘bushbabies’ OR ‘galago’ OR ‘galagos’ OR ‘anthropoidea’ OR ‘anthropoids’ OR ‘simian’ OR ‘simians’ OR ‘monkey’ OR ‘monkeys’ OR ‘marmoset’ OR ‘marmosets’ OR ‘callithrix’ OR ‘cebuella’ OR ‘tamarin’ OR ‘tamarins’ OR ‘saguinus’ OR ‘leontopithecus’ OR ‘squirrel monkey’ OR ‘squirrel monkeys’ OR ‘saimiri’ OR ‘night monkey’ OR ‘night monkeys’ OR ‘owl monkey’ OR ‘owl monkeys’ OR ‘douroucoulis’ OR ‘aotus’ OR ‘spider monkey’ OR ‘spider monkeys’ OR ‘ateles’ OR ‘baboon’ OR ‘baboons’ OR ‘papio’ OR ‘rhesus monkey’ OR ‘macaque’ OR ‘macaca’ OR ‘mulatta’ OR ‘cynomolgus’ OR ‘fascicularis’ OR ‘green monkey’ OR ‘green monkeys’ OR ‘chlorocebus’ OR ‘vervet’ OR ‘vervets’ OR ‘pygerythrus’ OR ‘hominoidea’ OR ‘ape’ OR ‘apes’ OR ‘hylobatidae’ OR ‘gibbon’ OR ‘gibbons’ OR ‘siamang’ OR ‘siamangs’ OR ‘nomascus’ OR ‘symphalangus’ OR ‘hominidae’ OR ‘orangutan’ OR ‘orangutans’ OR ‘pongo’ OR ‘chimpanzee’ OR ‘chimpanzees’ OR ‘pan troglodytes’ OR ‘bonobo’ OR ‘bonobos’ OR ‘pan paniscus’ OR ‘gorilla’ OR ‘gorillas’ OR ‘troglodytes’):ti,ab

## WEB OF SCIENCE

### SC1 Animal Welfare

TS=(animal welfare OR (animal AND welfare) OR animal wellbeing OR (animal AND wellbeing) OR animal well-being OR (animal AND well-being))

### SC2 Glucocorticoids

TS=(corticosterone OR corticosterone blood level OR corticosterone release OR corticosterone OR corticocorticosterone OR corticocosteroid OR corticorterone OR corticoserone OR corticostcrone OR corticosteone OR corticoster OR corticosterene OR corticostereone OR corticosteron OR cortikosterone OR cortikosteron OR kortikosterone OR kortikosteron OR kendall compound b OR reichstein substance h OR corticoesterone OR cortecosterone OR corticossterone OR cortcosterone OR cortisol OR cortisol levels OR cortisone OR cortizone OR corticosteroid OR corticosteroids OR adrenal cortical steroid OR corticoid OR corticoids OR cortef OR hydrocortone OR hydrocortisone OR glucocorticoids OR glucocorticoid OR glucocorticosteroid OR glucocorticosteroids)

### SC3 All Non-human animals excluding invertebrates

TS=(animal exp* OR exp* animal OR animal model* OR animal* OR animal population groups OR chordata OR vertebrates OR amphibia* OR tetrapods OR birds OR fishes OR reptiles OR mammal* OR primates OR artiodactyla OR carnivores OR cetacea OR chiroptera OR elephants OR hyraxes OR insectivores OR lagomorpha OR marsupialia OR monotremata OR pisces OR perissodactyla OR rodentia OR scandentia OR sirenia OR xenarthra OR haplorhini OR strepsirhini OR platyrrhini OR tarsii OR catarrhini OR cercopithecidae OR hylobatidae OR hominidae OR gorilla gorilla OR pan paniscus OR pan troglodytes OR pongo pygmaeus OR mice OR mus OR mouse OR murine OR woodmouse OR rats OR rat OR murinae OR muridae OR cottonrat OR cottonrats OR hamster OR hamsters OR cricetinae OR rodentia OR rodent OR rodents OR pigs OR pig OR swine OR swines OR piglets OR piglet OR boar OR boars OR sus scrofa OR ferrets OR ferret OR polecat OR polecats OR mustela putorius OR guinea pigs OR guinea pig OR cavia OR callithrix OR marmoset OR marmosets OR cebuella OR hapale OR octodon OR chinchilla OR chinchillas OR gerbillinae OR gerbil OR gerbils OR jird OR jirds OR merione OR meriones OR rabbits OR rabbit OR hares OR hare OR diptera OR flies OR fly OR dipteral OR drosophila OR drosophilidae OR cats OR cat OR carus OR felis OR nematoda OR nematode OR nematodes OR sipunculida OR dogs OR dog OR canine OR canines OR canis OR sheep OR sheeps OR mouflon OR mouflons OR ovis OR goats OR goat OR capra OR capras OR rupicapra OR rupicapras OR chamois OR haplorhini OR monkey OR monkeys OR anthropoidea OR anthropoids OR saguinus OR tamarin OR tamarins OR leontopithecus OR ape OR apes OR pan paniscus OR bonobo OR bonobos OR gibbon OR gibbons OR siamang OR siamangs OR nomascus OR symphalangus OR chimpanzee OR chimpanzees OR prosimian OR prosimians OR bush baby OR bush babies OR galagos OR galago OR pongidae OR gorilla OR gorillas OR pongo pygmaeus OR orangutan OR orangutans OR lemur OR lemurs OR lemuridae OR horse OR horses OR equus OR cow OR calf OR bull OR avian OR avians OR chicken OR chickens OR gallus OR quail OR duck OR goose OR geese OR turkey OR bird OR birds OR quails OR poultry OR poultries OR fowl OR fowls OR reptile OR reptilia OR reptiles OR snakes OR snake OR lizard OR lizards OR alligator OR alligators OR crocodile OR crocodiles OR turtle OR turtles OR amphibian OR amphibians OR amphibia OR frog OR frogs OR bombina OR salientia OR toad OR toads OR epidalea calamita OR salamander OR salamanders OR eel OR eels OR fish OR fishes OR pisces OR catfish OR catfishes OR siluriformes OR arius OR heteropneustes OR sheatfish OR perch OR perches OR percidae OR perca OR trout OR trouts OR char OR chars OR salvelinus OR minnow OR cyprinidae OR carps OR carp OR zebrafish OR zebrafishes OR goldfish OR goldfishes OR guppy OR guppies OR chub OR chubs OR tinca OR barbels OR barbus OR pimephales OR promelas OR poecilia reticulata OR mullet OR mullets OR seahorse OR seahorses OR mugil curema OR atlantic cod OR shark OR sharks OR catshark OR anguilla OR salmonid OR salmonids OR whitefish OR whitefishes OR salmon OR salmons OR sole OR solea OR lamprey OR lampreys OR pumpkinseed OR sunfish OR sunfishes OR tilapia OR tilapias OR turbot OR turbots OR flatfish OR flatfishes OR sciuridae OR squirrel OR squirrels OR chipmunk OR chipmunks OR suslik OR susliks OR vole OR voles OR lemming OR lemmings OR muskrat OR muskrats OR lemmus OR otter OR otters OR marten OR martens OR martes OR weasel OR badger OR badgers OR ermine OR mink OR minks OR sable OR sables OR gulo OR gulos OR wolverine OR wolverines OR mustela OR llama OR llamas OR alpaca OR alpacas OR camelid OR camelids OR guanaco OR guanacos OR chiroptera OR chiropteras OR bat OR bats OR fox OR foxes OR iguana OR iguanas OR xenopus laevis OR parakeet OR parakeets OR parrot OR parrots OR donkey OR donkeys OR mule OR mules OR zebra OR zebras OR shrew OR shrews OR bison OR bisons OR buffalo OR buffaloes OR deer OR deers OR bear OR bears OR panda OR pandas OR wild hog OR wild boar OR fitchew OR fitch OR beaver OR beavers OR jerboa OR jerboas OR capybara OR capybaras OR bovine OR porcine OR hog OR hogs OR catfish OR catfishes OR sheatfish OR silurus OR arius OR heteropneustes OR clarias OR gariepinus OR fathead minnow OR fathead minnows OR pimephales OR promelas OR cichlidae OR trout OR trouts OR char OR chars OR salvelinus OR salmo OR oncorhynchus OR guppy OR guppies OR millionfish OR poecilia OR goldfish OR goldfishes OR carassius OR auratus OR mullet OR mullets OR mugil OR curema OR shark OR sharks OR cod OR cods OR gadus OR morhua OR carp OR carps OR cyprinus OR carpio OR killifish OR anguilla OR zander OR sander OR lucioperca OR stizostedion OR turbot OR turbots OR psetta OR flatfish OR flatfishes OR plaice OR pleuronectes OR platessa OR tilapia OR tilapias OR oreochromis OR sarotherodon OR common sole OR dover sole OR solea OR zebrafish OR zebrafishes OR danio OR rerio OR seabass OR dicentrarchus OR labrax OR morone OR lamprey OR lampreys OR petromyzon OR pumpkinseed OR pumpkinseeds OR lepomis OR gibbosus OR herring OR clupea OR harengus OR anura OR salientia OR frog OR frogs OR rana OR toad OR toads OR bufo OR xenopus OR laevis OR bombina OR epidalea OR calamita OR salamander OR salamanders OR newt OR newts OR triturus OR reptilia OR reptile OR reptiles OR bearded dragon OR pogona OR vitticeps OR iguana OR iguanas OR lizard OR lizards OR anguis fragilis OR turtle OR turtles OR snakes OR snake OR aves OR bird OR birds OR quail OR quails OR coturnix OR bobwhite OR colinus OR virginianus OR poultry OR poultries OR fowl OR fowls OR chicken OR chickens OR gallus OR zebra finch OR taeniopygia OR guttata OR canary OR canaries OR serinus OR canaria OR parakeet OR parakeets OR grasskeet OR parrot OR parrots OR psittacine OR psittacines OR shelduck OR tadorna OR branta OR leucopsis OR woodlark OR lullula OR flycatcher OR ficedula OR hypoleuca OR dove OR doves OR geopelia OR cuneata OR greylag OR graylag OR anser OR harrier OR circus pygargus OR red knot OR great knot OR calidris OR canutus OR godwit OR limosa OR lapponica OR meleagris OR gallopavo OR jackdaw OR corvus OR monedula OR ruff OR philomachus OR pugnax OR lapwing OR peewit OR plover OR vanellus OR swan OR cygnus OR columbianus OR bewickii OR gull OR chroicocephalus OR ridibundus OR albifrons OR great tit OR parus OR aythya OR fuligula OR streptopelia OR risoria OR spoonbill OR platalea OR leucorodia OR blackbird OR turdus OR merula OR blue tit OR cyanistes OR pigeon OR pigeons OR columba OR pintail OR anas OR starling OR sturnus OR owl OR athene noctua OR pochard OR ferina OR cockatiel OR nymphicus OR hollandicus OR skylark OR alauda OR tern OR sterna OR teal OR crecca OR oystercatcher OR haematopus OR ostralegus OR shrew OR shrews OR sorex OR araneus OR crocidura OR russula OR european mole OR talpa OR chiroptera OR bat OR bats OR eptesicus OR serotinus OR myotis OR dasycneme OR daubentonii OR pipistrelle OR pipistrellus OR cat OR cats OR felis OR catus OR feline OR dog OR dogs OR canis OR otter OR otters OR lutra OR badger OR badgers OR meles OR fitchew OR fitch OR foumart or foulmart OR ferrets OR ferret OR polecat OR polecats OR mustela OR putorius OR weasel OR weasels OR fox OR foxes OR vulpes OR common seal OR phoca OR vitulina OR grey seal OR halichoerus OR horse OR horses OR equus OR equine OR equidae OR donkey OR donkeys OR mule OR mules OR boar OR boars OR porcine OR sus OR scrofa OR llama OR llamas OR lama OR glama OR deer OR deers OR cervus OR elaphus OR cow OR cows OR bos taurus OR bos indicus OR bovine OR bull OR bulls OR cattle OR bison OR bisons OR sheep OR sheeps OR ovis aries OR ovine OR lamb OR lambs OR mouflon OR mouflons OR goat OR goats OR capra OR caprine OR chamois OR rupicapra OR leporidae OR lagomorpha OR lagomorph OR rabbit OR rabbits OR oryctolagus OR cuniculus OR laprine OR hares OR lepus OR murinae OR mouse OR mice OR mus OR musculus OR murine OR woodmouse OR apodemus OR rat OR rats OR rattus OR norvegicus OR cavia OR porcellus OR hamster OR hamsters OR mesocricetus OR cricetulus OR cricetus OR gerbil OR gerbils OR jird OR jirds OR meriones OR unguiculatus OR jerboa OR jerboas OR jaculus OR chinchilla OR chinchillas OR beaver OR beavers OR castor fiber OR castor canadensis OR sciuridae OR sciurus OR chipmunk OR chipmunks OR marmot OR marmots OR marmota OR suslik OR susliks OR spermophilus OR cynomys OR cottonrat OR cottonrats OR sigmodon OR vole OR voles OR microtus OR myodes OR glareolus OR primate OR primates OR prosimian OR prosimians OR lemur OR lemurs OR lemuridae OR loris OR bushbaby OR bushbabies OR galago OR galagos OR anthropoidea OR anthropoids OR simian OR simians OR marmoset OR marmosets OR callithrix OR cebuella OR tamarin OR tamarins OR saguinus OR leontopithecus OR saimiri OR douroucoulis OR aotus OR ateles OR baboon OR baboons OR papio OR macaque OR macaca OR mulatta OR cynomolgus OR fascicularis OR chlorocebus OR vervet OR vervets OR pygerythrus OR hominoidea OR ape OR apes OR hylobatidae OR gibbon OR gibbons OR siamang OR siamangs OR nomascus OR symphalangus OR orangutan OR orangutans OR pongo OR chimpanzee OR chimpanzees OR bonobo OR bonobos OR pan paniscus OR troglodytes)
